# Low-intensity logging and hunting have long-term effects on seed dispersal but not fecundity in Afrotropical forests

**DOI:** 10.1093/aobpla/ply074

**Published:** 2018-12-13

**Authors:** Chase L Nuñez, James S Clark, Connie J Clark, John R Poulsen

**Affiliations:** 1 University Program in Ecology, Duke University, Durham, USA; 2 Nicholas School of the Environment, Duke University, Durham, USA

**Keywords:** Anthropocene, dispersal, frugivory, habitat fragmentation, hunting, selective logging, tropical forest

## Abstract

Hunting and logging, ubiquitous human disturbances in tropical forests, have the potential to alter the ecological processes that govern population recruitment and community composition. Hunting-induced declines in populations of seed-dispersing animals are expected to reduce dispersal of the tree species that rely on them, resulting in potentially greater distance- and density-dependent mortality. At the same time, selective logging may alter competitive interactions among tree species, releasing remaining trees from light, nutrient or space limitations. Taken together, these disturbances may alter the community composition of tropical forests, with implications for carbon storage, biodiversity conservation and ecosystem function. To evaluate the effects of hunting and logging on tree fecundity and seed dispersal, we use 3 years of seed rain data from a large-scale observational experiment in previously logged, hunted and protected forests in northern Republic of Congo (Brazzaville). We find that low-intensity logging had a meaningful long-term effect on species-specific seed dispersal distances, though the direction and magnitude varied and was not congruent within dispersal vector. Tree fecundity increased with tree diameter, but did not differ appreciably across disturbance regimes. The species-specific dispersal responses to logging in this study point towards the long-lasting toll of disturbance on ecological function and highlight the necessity of conserving intact forest.

## Teaser

Hunting and logging can alter ecological communities, but their long-term effects are seldom studied in Afrotropical forests. Hunting-related declines in seed-dispersing animal populations are expected to reduce dispersal of the tree species that rely on them, resulting in fewer successful offspring. At the same time, selective logging may alter competitive interactions among species, releasing remaining trees from light, nutrient or space limitations. We find that low-intensity logging affected seed dispersal two decades after the logging event. The difficult-to-detect effects on a key ecological process could have direct consequences for the diversity and function of forest ecosystems.

## Introduction

Logging concessions now cover almost 56 million ha of forest in West and Central Africa (FAO 2016). Most concessions are subject to low-intensity, selective logging intended to reduce the negative ecological impacts of traditional, conventional logging operations. Studies across the tropics have demonstrated that selective logging techniques can substantially reduce the short-term effects of logging (Sist 2000; Sist *et al.* 2003; Medjibe *et al.* 2013), but few studies have considered the long-term effects of selective logging on critical forest processes (Brown and Gurevitch 2004; Meijaard *et al.* 2005). Tropical trees respond to environmental disturbance on timescales that usually surpass the duration of ecological studies (Gourlet-Fleury *et al.* 2013; Edwards *et al.* 2014; Berdanier and Clark 2015) and changes in tree fecundity and seed dispersal may persist long after disturbance has ended, potentially altering ecosystem function.

Logging directly disturbs tropical forest communities through the extraction of large trees (Laurance *et al.* 2000), residual damage to remaining trees (Kasenene and Murphy 1991) and disruption of seed-dispersing animal communities (Gutiérrez-Granados 2011; Haurez *et al.* 2016; Rosin and Poulsen 2016). Road construction fragments the forest and provides hunters access to previously inaccessible areas (Kleinschroth and Healey 2017). Unsustainable hunting is the major cause of defaunation in many parts of the world (Hoffmann *et al.* 2010), causing over a quarter of the world’s vertebrate species to decline in abundance over the last four decades (Dirzo *et al.* 2014). Reductions in vertebrate dispersers may affect the approximately two-thirds of all woody plants that rely on animals for seed dispersal (Willson and Traveset 2000; Muller-Landau and Hardesty 2005; Beaune *et al.* 2013). Dispersal failure has consequences for community composition through density-dependent recruitment (Cannon *et al.* 1994; Bleher and Böhning-Gaese 2001) and competition at later life stages (Nathan and Muller-Landau 2000).

Studies investigating how hunting and logging affect seed dispersal have yielded mixed results (Theimer *et al.* 2011; Beck *et al.* 2013; Kurten 2013; Camargo-Sanabria *et al.* 2014; Comita *et al.* 2014; Rosin and Poulsen 2016) in part because the interacting effects of hunting and logging have not been quantified beyond their immediate responses to disturbances (Markl *et al.* 2012). In the short term, intermediate levels of disturbance from selective logging may increase light and nutrients available to survivors (Johns 1988; Kasenene and Murphy 1991; Cannon *et al.* 1994; Huante *et al.* 1998; John *et al.* 2007; Ewel and Mazzarino 2008; Gutiérrez-Granados 2011; Haurez *et al.* 2016), thereby increasing tree fecundity (Molino and Sabatier 2001; Clark *et al.* 2010, 2014b). Logging may even increase the dispersal distance of abiotically dispersed species following forest thinning due to greater wind speeds through the canopy (Gardiner 1994; Stacey *et al.* 1994; Gardiner *et al.* 1997). However, in the longer term, logging may reduce seed dispersal distance and fecundity through combinations of increased hunting pressure (Kleinschroth and Healey 2017), declines in vertebrate dispersal vectors (Poulsen *et al.* 2013; Haurez *et al.* 2016), soil compaction (Pinard *et al.* 2000) and invasion of fast-growing competitors (Schnitzer and Bongers 2002). Because declines in dispersal vectors and increases in fecundity can both follow disturbance, investigating the interactions of these processes is essential for understanding the underlying ecological process (Abernethy *et al.* 2013).

To evaluate the separate and combined effects of hunting and logging on both fecundity and dispersal for animal and abiotically dispersed trees, we collected 3 years of seed rain data from a large-scale observational experiment in previously logged, hunted and protected forests in northern Republic of Congo (Brazzaville). By controlling for logging and hunting in our sampling design, we offer a first opportunity to test their relative effects. We hypothesized that the fecundity and dispersal distances of tropical trees will be sensitive to both hunting and logging. Specifically, we expected that: (i) tree fecundity is greater in logged forests relative to protected forests, regardless of whether trees species are abiotically or animal dispersed; and (ii) hunting reduces dispersal distances of animal-dispersed species, but not the dispersal distances of abiotically (wind or ballistic) dispersed species. Understanding the separate and combined effects of disturbances on seed dispersal is critical to predict changes in forest species composition and diversity.

## Materials and Methods

### Study area

**Figure 1.**
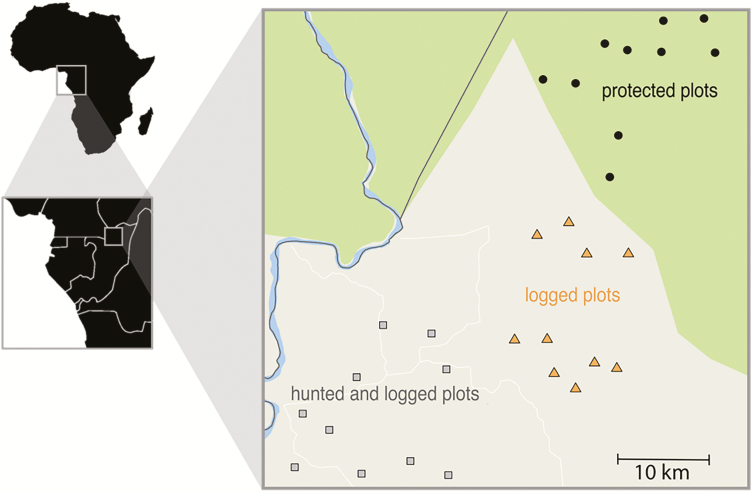
Location of 30 1-ha study plots in Northern Congo. Protected plots fall within the border of Nouabale-Ndoki National Park (green), whereas plots exposed to hunting and/or logging were located in the Kabo logging concession (grey) in northern Republic of Congo.

We conducted the study in the Nouabale Ndoki National Park (NNNP; 400 000 ha) and the Kabo logging concession (267 000 ha) in northern Republic of Congo (Fig. 1). The forests in this area are classified as lowland tropical forest. Dominant tree families include *Meliaceae*, *Euphorbiaceae* and *Annonaceae* (CIB 2006). Rainfall averages ~1700 mm annually and is seasonal with peaks in May and October. The Kabo concession borders the NNNP to the south, and together they include a mosaic of logged and unlogged forest. Twenty years before the study began, the logging concession was selectively logged at low intensity (<2.5 stems per hectare) with four species, *Entandophragma cylindricum*, *E. utile*, *Triplochiton scleroxylon* and *Milicia excelsa*, making up 90 % of the harvest volume (CIB 2006). Although we do not have data on rates of natural disturbance at our study site, a comparison of pantropical data (*n* = 65) report a range of natural stand mortality from 0.86 to 2.02 %, with a best estimate of adjusted stem turnover rate of 1.81 ± 0.16 % (Lewis *et al.* 2004). Approximately 3000 people inhabited the study site at the time of the study, most residing in the logging town of Kabo. Residents generally hunted with shotguns, and to a lesser extent with wire snares, for consumption and for local trade (Poulsen *et al.* 2009). A gradient of hunting intensity decreases with distance from Kabo, with some forest types being used more than others (Mockrin 2008).

### Tree census and seed rain data

We established 30 1-ha tree plots comprised of three equal-area groups, including 10 sites that were unlogged and unhunted, 10 sites that were logged and unhunted and 10 sites that were both logged and hunted. Using ArcView 3.2 and a 14-class habitat map (Laporte *et al.* 2007), we randomly located plots within each disturbance regime in mixed lowland forest, with a buffer of at least 500 m to the nearest primary road and 100 m to the nearest water source. Within each plot, all trees >10 cm diameter at breast height (DBH) were tagged, measured, mapped and identified to species (Wortley and Harris 2014). We additionally recorded canopy status (understory, midstory, canopy and emergent) and presence of lianas in the crown. Canopy openness and light availability were estimated for each plot by averaging values from four hemispherical pictures taken at each quarter of a plot. Seed traps 1 m^2^ in area were centred along three transects at 25, 50 and 75 m from a plot border, with 10 m separating each trap. All traps were at least 20 m from the nearest plot border. Seeds and fruits were collected every 2 weeks and identified to species or genus level. Previous evidence demonstrates that parameter estimates are dominated by the relatively abundant seeds falling from within these distances (Clark *et al.* 1998).

We used seed rain data from 33 of the most common species to quantify fecundity and seed dispersal dynamics. Although seed rain was collected on many more species, we limited analysis to species that occurred in at least half of all plots. Tree density, size and species composition were approximately equivalent across plots and disturbance types **[see****Supporting Information—Figs S1**–**S3****]**. Of the 44 species that contributed seeds to at least half of the plots, 11 were lianas—woody vines that rely on trees for support. We omitted liana species from the present study despite their clear importance for frugivore diets, because they extend laterally tens of metres from their rooting stems, making the attribution of seeds to a censused stem challenging. The number of focal trees per 1-ha plot ranged from 50 to 253 with a median of 155 trees, and the number of seeds per focal species per plot ranged from 16 to 288 with a median of 96.

### Plant species trait data

The dispersal mode for each tree species was assigned based on fruit morphology and observations of fruit consumption (Gautier-Hion *et al.* 1985; Tutin *et al.* 1997; White and Abernethy 1997; Whitney *et al.* 1998; Clark *et al.* 2001; Poulsen *et al.* 2001, 2002; Hawthorne and Gyakari 2006; Morgan and Sanz 2006) **[see****Supporting Information—Table S1****]**. Because many animal-dispersed species are dispersed by both birds and mammals, we report results by broad classes of animal and abiotic (wind or ballistic) dispersal mode. In addition to dispersal mode, the mean tree DBH (cm) and tree density (stems per hectare) for each species were also calculated by forest type to relate dispersal parameters to species characteristics.

### Fecundity estimation and dispersal analysis

**Figure 2.**
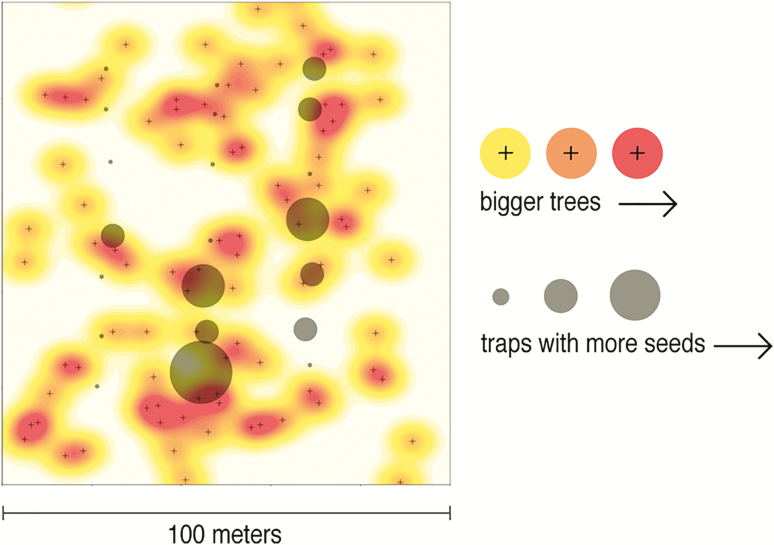
A schematic of seed shadow modelling, with spatially distributed trees of varying sizes acting as signal sources of varying strengths, and seed traps acting as stationary detectors through time.

We use a state-space model for Mast Inference and Forecasting (available on CRAN as the R package MASTIF, http://rpubs.com/jimclark/281413) to determine the relative influence of hunting and logging on the fecundity and dispersal kernel of each tree (Clark, Nuñez and Tomasek, in revision). Mast Inference and Forecasting builds on the rich literature of seed dispersal models that employ a bivariate Student’s t (2Dt) to relate the size and locations of reproductively active trees to numbers of seeds collected in seed traps in order to probabilistically estimate the seed production of each tree (Fig. 2; Clark *et al.* 1999, 2010, 2014a). Some authors use a two-parameter version of the 2Dt kernel; we do not fit a shape parameter due to the fact that it is poorly identified in data and it does not respond to the tail of the kernel as was originally hoped (e.g. Clark *et al.* 1999).

Not all seeds in seed traps must come from trees within the inventory plot. This possibility suggests an intercept proportional to basal area (Clark *et al.* 2010) or an integral over a large landscape area (Muller-Landau *et al.* 2008) as a rough accommodation of long-distance dispersal. In our comparisons an intercept can change estimates, without actually being sensitive to seeds outside the plot. This insensitivity to distant trees was demonstrated by Clark *et al.* (1998) by fitting the model without intercept to increasingly expanded plot areas. An intercept is insensitive to long-distance dispersal because distant trees do not affect the likelihood; the tail of the kernel has no impact on estimates except in cases where seeds are rare (Clark *et al.* 1999). The converse is also true: standard errors on estimates of fecundity increase with distance from seed traps. The intercept model further requires a strict assumption about forest composition outside the plot, e.g. extrapolating composition within the plot to infinite distance (Muller-Landau *et al.* 2008; Clark *et al.* 2010), which is unrealistic in many forests.

Mast Inference and Forecasting extends the model that has been extensively tested with predictive distributions to allow for uncertainty in seed identification, as well as time-dependence (Clark *et al.* 2004, 2010) and quasi-periodic variation and synchronicity in seed production (Koenig and Knops 2001; Boutin *et al.* 2006; Wang *et al.* 2017). Mast Inference and Forecasting uses Gibbs sampling—a Markov chain Monte Carlo (MCMC) technique—as well as Metropolis and Hamiltonian Markov chain (HMC) for posterior simulations of tree maturation state, fecundity, seed dispersal kernel and parameter estimates. Parameter estimates—the effects of hunting, logging and site-level covariates—are sampled directly from the posterior (Clark, Nuñez and Tomasek, in revision). We used non-informative flat priors for the dispersal parameter and variance in the dispersal parameter with fixed degrees of freedom as detailed in Clark *et al.* (2004, 2010, 2014a).

The broad dispersion of seed count data is accommodated in at least one of two ways. If accommodated at the data stage with a negative binomial distribution (Clark *et al.* 1998; Muller-Landau *et al.* 2008), then the dispersion parameter has no biological interpretation, and it cannot respond to the variables that are known to affect seed variability. Alternatively, a hierarchical specification helps to explain that variation, through individual differences in covariates and random effects and year or lag effects (Clark *et al.* 2004, 2013; Martínez and González-Taboada 2009; Uriarte *et al.* 2012). In other words, the overdispersion is taken up by the underlying process; the data are conditionally Poisson, but marginally overdispersed (Clark, Nuñez and Tomasek, in revision). Our model incorporates a Poisson likelihood for count data with seed production and dispersal, written as:

$$\begin{array}{l} E(y_{s})=A\lambda_{s}=A\underset{i=1}{\overset{n}{\sum }}\;S_{si}f_{i} \end{array}$$

where *E*(*y*_*s*_) is the expected number of seeds counted in a trap at location *s. λ*_*s*_ is the expected seed density (seeds per m^2^ per year) multiplied by the sampling effort *A*—the area of a seed trap times the fraction of the fruiting season it was deployed (m^2^ per year). *S*_*si*_ is the density of seed (m^−2^) produced by tree *i* dispersed to seed trap location *s*; and *f*_*i*_ is fecundity for an individual tree *i* at time *t*, which is the product of maturation status (*ρ*_*it*_) and conditional fecundity (*ψ*_*it*_) of tree *i*, (*f*_*i,t*_) = *ψ*_*i,t*_*ρ*_*i,t*_ ≥ 0. Maturation and conditional fecundity are dynamic processes, modelled with fixed, random and year effects. Coefficients in the vector of fixed effects *β*^*x*^ include tree diameter, exposure to hunting or logging, and interactions (Clark 2010; Clark *et al.* 2013). Random individual effects accommodate the heterogeneity of responses among individual trees. The effect of year is random across species and within each of the three disturbance types, accommodating seed rain fluctuations that are coherent within, but not among the three groups.

Dispersal is summarized by the mean parameter of the 2Dt dispersal kernel (Clark *et al.* 1999), here termed the ‘dispersal parameter’. A shape parameter is also sometimes fitted for this model, but we have found it to be unstable and unresponsive to long-distance dispersal (Clark *et al.* 2004, 2010).

Our modelling did not explicitly incorporate boundary effects because previous analysis demonstrated that trees tens of metres from seed traps have little impact on estimates (Clark *et al.* 1999). Muller-Landau *et al.* (2008), however, concluded that failure to account for boundary effects could bias models towards higher fecundity and fat tails (Muller-Landau *et al.* 2008), leading to overestimated fecundities and dispersal distances. However, this would not change inferences related to the relative effects of vectors or disturbance on seed dispersal patterns.

Gibbs sampling was used for posterior simulation. For each tree species **[see****Supporting Information—Fig. S5****]**, model estimates were taken from 50 000 iterations, discarding the first 1000 iteration as pre-convergence. We visually inspected trace plots to confirm convergence and adequate mixing **[see****Supporting Information—Fig. S6A**–**C****]**. Model fit was assessed with root mean squared prediction error (RMSPE) across species **[see****Supporting Information—Fig. S4****]**. Variable selection was based on Deviance Information Criterion (DIC). Model estimates reported in the text are posterior means and 95 % credible intervals (CIs) based on the Gibbs sampler realizations.

## Results

Hunting and logging influenced the mean distances of dispersal kernels (hereafter average dispersal distance), with the greatest effects on animal-dispersed species, though the direction and magnitude varied. Two-thirds of all species (22/33) in disturbed forests had 95 % CIs for dispersal parameters that did not overlap with estimates from protected plots, indicating a role of disturbance. This trend held true whether a species relied on animals for dispersal entirely (13/18), in part (5/8) or not at all (4/7).

Of the 22 species affected by disturbance, 17 species showed an effect of logging alone: nine species had higher dispersal estimates in logged compared to protected forest (*Celtis mildbraedii*, *Diospyros canaliculata*, *Erythrophleum suaveolens*, *Greenwayodendron suaveolens*, *Lannea welwitschii*, *Pausinystalia macroceras*, *Rinorea oblongifolia*, *Staudtia kamerunensis*, *Strombosia nigropunctata*), and eight species had lower dispersal estimates (*Cleistopholis patens*, *Grossera macrantha*, *Myrianthus arboreus*, *Macaranga barteri*, *Nesogordonia kabingaensis*, *Strombosiopsis tetrandra*, *Thomandersia hensii*, *Terminalia superba*).

**Figure 3.**
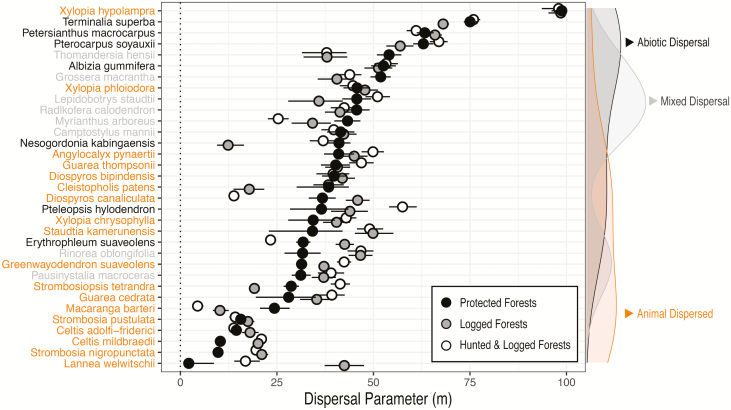
Comparison of average dispersal distance parameters among species in plots that were hunted and logged, logged, or protected from hunting and logging. Species are ordered by mean dispersal distance parameter in protected plots. Densities on right *Y*-axis show distribution of the dispersal type for species on left *Y*-axis.

**Figure 4.**
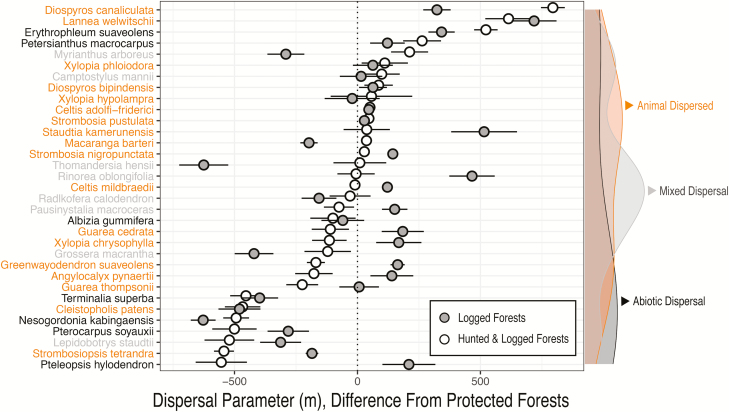
Comparison of difference in average dispersal distance parameter from protected forests among species in plots that were hunted and logged, or logged. Species are ordered by mean dispersal distance in hunted and logged plots. Densities on right *Y*-axis show distribution of the dispersal type for species on left *Y*-axis.

The combined effects of hunting and logging were consistent with logging alone for the majority of species, with the exception of six species that had dispersal estimates greater than (*Pteleopsis hylodendron*, *S. tetrandra*, *Guarea cedrata*) or less than (*G. macrantha*, *D. canaliculata* and *E. suaveolens*) logging alone. Notably, three species exhibited divergent effects of disturbance regime on dispersal estimates: logging positively affected *D. canaliculata* and *E. suaveolens*, whereas the combination of hunting and logging negatively affected dispersal estimates relative to protected plots. *Strombosiopsis tetrandra* displayed the opposite pattern (Table 1; Figs 3 and 4).

**Table 1.** Predictive mean and 95 % CI for seed dispersal distances in metres.

	Mean predicted dispersal distance								
	Logged forests			Protected forests			Hunted and logged forests		
	Estimate	2.50 %	97.50 %	Estimate	2.50 %	97.50 %	Estimate	2.50 %	97.50 %
Abiotically dispersed									
*Albizia gummifera*	51.3	47.7	54.9	52.6	49.4	55.8	53.3	50.3	56.3
*Erythrophleum suaveolens*	42.6	40.2	45.0	31.8	30.0	33.7	23.4	22.1	24.7
*Nesogordonia kabingaensis*	12.4	9.5	16.5	41.1	38.1	44.1	37.0	33.5	40.6
*Petersianthus macrocarpus*	65.9	64.1	67.7	63.3	61.2	65.6	61.1	58.6	63.4
*Pteleopsis hylodendron*	43.9	39.0	48.6	36.5	28.4	43.6	57.5	54.0	61.2
*Pterocarpus soyauxii*	56.9	53.3	60.4	62.9	60.3	65.5	66.9	64.6	69.2
*Terminalia superba*	68.0	66.6	69.6	75.0	73.4	76.8	75.9	74.3	77.7
									
Animal dispersed									
*Angylocalyx pynaertii*	45.0	41.5	48.5	41.0	37.2	45.0	49.9	46.9	52.7
*Celtis adolfi-friderici*	18.0	16.0	20.3	14.5	13.2	15.9	13.8	12.9	14.8
*Celtis mildbraedii*	20.1	18.7	21.6	10.3	9.9	10.8	21.0	19.8	22.3
*Cleistopholis patens*	17.8	13.8	21.7	38.4	30.1	43.6	38.4	34.4	42.3
*Diospyros bipindensis*	41.9	38.7	45.2	39.8	36.7	43.1	39.5	35.2	43.8
*Diospyros canaliculata*	45.9	42.8	49.0	36.8	33.2	40.2	13.9	12.8	15.0
*Greenwayodendron suaveolens*	37.2	35.7	38.7	31.4	30.2	32.7	42.4	40.5	44.2
*Guarea cedrata*	35.3	31.0	39.7	28.0	19.5	35.1	39.2	35.9	42.6
*Guarea thompsonii*	40.7	37.5	44.0	40.2	36.4	43.9	46.9	43.7	50.1
*Lannea welwitschii*	42.5	37.3	47.6	2.2	1.0	8.7	16.9	14.0	20.6
*Macaranga barteri*	10.2	8.4	12.5	24.4	20.6	28.3	4.5	3.6	6.0
*Staudtia kamerunensis*	49.9	45.2	55.2	34.2	22.9	42.0	49.0	45.7	52.5
*Strombosia nigropunctata*	21.1	19.5	22.8	9.8	9.1	10.5	19.6	18.3	21.0
*Strombosia pustulata*	17.5	15.9	19.2	15.6	14.5	16.9	14.2	12.7	15.9
*Strombosiopsis tetrandra*	19.2	18.1	20.3	28.7	26.7	30.7	41.4	38.9	43.9
*Xylopia chrysophylla*	40.4	36.9	44.0	34.4	27.9	40.0	42.9	40.3	45.6
*Xylopia hypolampra*	98.5	95.3	100.0	98.8	96.1	100.0	98.0	93.6	99.9
*Xylopia phloiodora*	47.8	44.3	51.2	45.7	42.2	49.2	44.7	41.3	48.1
									
Abiotic and animal dispersed									
*Camptostylus mannii*	42.3	38.9	45.6	41.5	38.0	45.1	39.7	36.5	43.0
*Grossera macrantha*	40.5	35.6	45.0	51.9	49.2	54.5	43.9	40.1	46.9
*Lepidobotrys staudtii*	35.9	27.9	42.1	45.7	41.9	49.4	51.1	47.9	54.3
*Myrianthus arboreus*	34.2	28.8	39.0	43.3	39.8	46.6	25.3	22.7	28.1
*Pausinystalia macroceras*	37.1	34.0	40.3	31.2	28.8	33.7	39.0	35.6	42.4
*Radlkofera calodendron*	41.3	37.4	45.1	45.7	42.1	49.0	42.4	38.9	46.0
*Rinorea oblongifolia*	46.7	43.6	49.8	31.7	27.0	36.3	46.7	43.3	50.0
*Thomandersia hensii*	38.0	31.9	43.2	54.0	50.9	57.2	37.9	31.5	43.1

**Figure 5.**
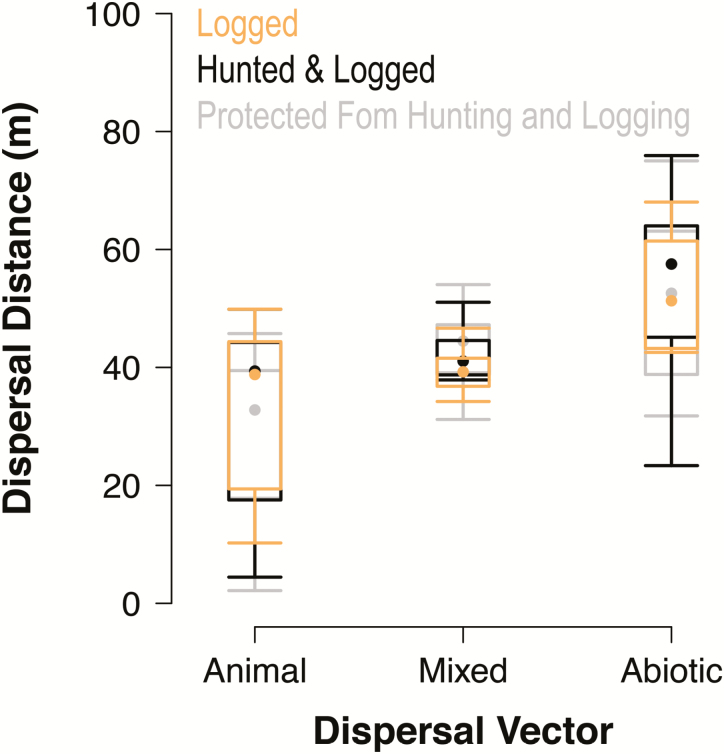
*Post hoc* comparison of the mean predicted dispersal distances for all species grouped by dispersal vector. Error bars show the 2.5th and 97.5th quantiles of mean dispersal distance in forests that were logged, hunted and logged, or protected from hunting and logging.

To reveal potential group-level effects of dispersal vectors, we clustered dispersal parameters from individual species by dispersal vector (i.e. animal, abiotic or mixed dispersal). Predictions were congruent within each dispersal vector, regardless of disturbance type (Fig. 5). Abiotically dispersed species had the greatest dispersal estimates overall, with 51.4 m [2.5th and 97.5th quantiles: 17.9, 75.5]. Species dispersed both by animals and abiotically had dispersal estimates of 41.1 m [28.7, 52.8], and animal-dispersed species had the lowest dispersal estimates of 34.4 m [6.2, 98.3].

To evaluate the group-level effects of disturbance, we clustered dispersal estimates of all species by disturbance type, including protected (38.9 m [8.2, 79.8]), hunted and logged (40.5 m [12.0, 80.3]) and logged forests (39.6 m [11.9, 74.1]). The large overlap in dispersal estimates among forest types indicates a lack of consistent effects of disturbance on dispersal distance.

**Figure 6.**
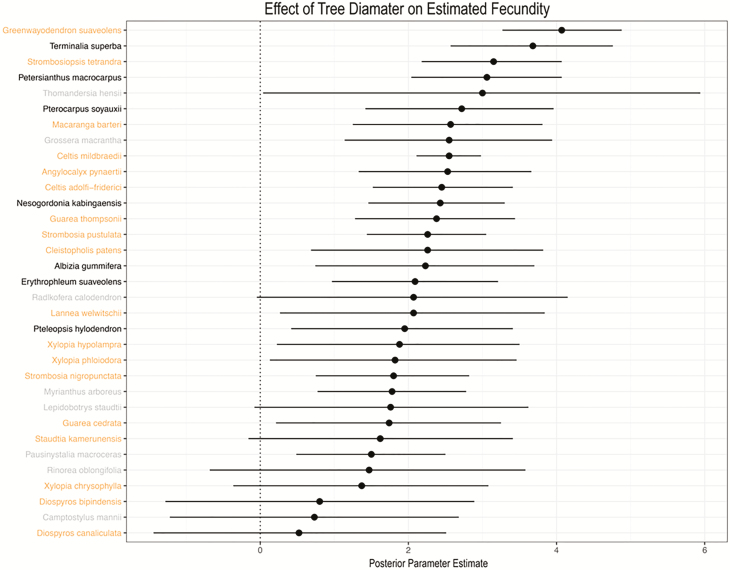
Comparison of posterior parameter estimates and 95 % CI show a positive effect of tree diameter on tree fecundity for a majority of species. Species names are colour coordinated here as elsewhere in the manuscript to denote dispersal vector: animal dispersed (orange), abiotically dispersed (black) or both animal and abiotically dispersed (grey).

**Figure 7.**
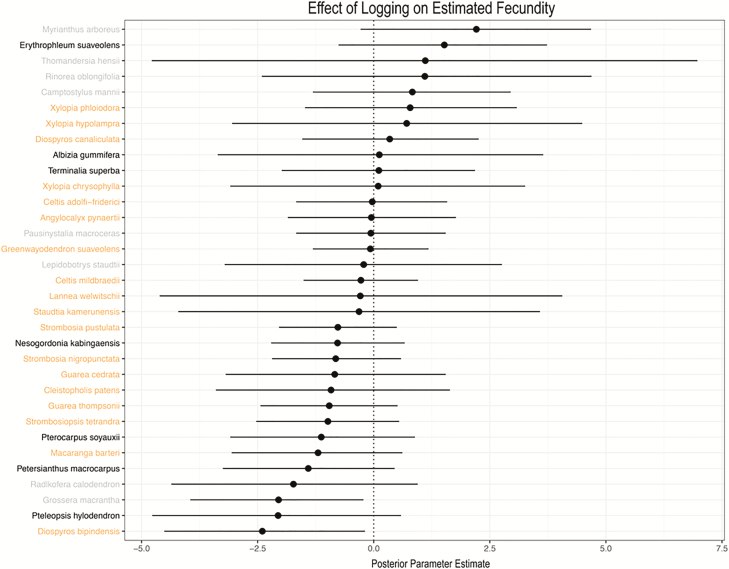
Comparison of posterior parameter estimates and 95 % CI show no effect of logging on tree fecundity for a majority of species.

**Figure 8.**
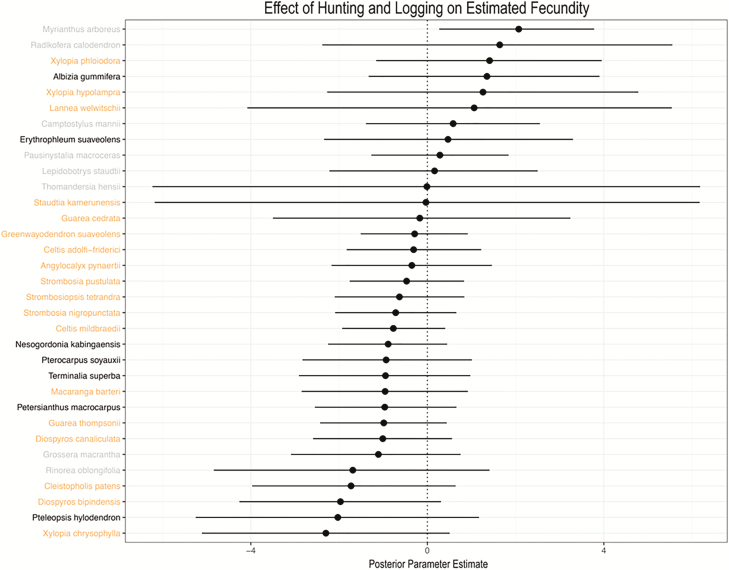
Comparison of posterior parameter estimates and 95 % CI show no effect of hunting and logging on tree fecundity for a majority of species.

Estimated tree fecundity increased with tree diameter (Fig. 6), but was not affected by disturbance regime (Table 2; Figs 7 and 8). A majority of species (25/33) exhibited a positive effect of tree diameter on fecundity, with the exception of *Radlkofera calodendron*, *Lepidobotrys staudtii*, *S. kamerunensis*, *R. oblongifolia*, *Xylopia chrysophylla*, *Diospyros bipindensis*, *Camptostylus mannii* and *D. canaliculata*. Logging only influenced fecundity estimates of three species (*D. bipindensis*, posterior mean and 95 % CIs: −1.78 [−3.51, −0.03], *G. macrantha* −1.49 [−2.96, −0.08] and *M. arboreus* 2.30 [0.67, 3.84]).

**Table 2.** Posterior mean and 95 % CIs of covariate effects on conditional fecundity.

	Covariate effects on conditional fecundity								
	Diameter			Logging			Hunting and logging		
	Posterior mean	2.50 %	97.50 %	Posterior mean	2.50 %	97.50 %	Posterior mean	2.50 %	97.50 %
Abiotically dispersed									
*Albizia gummifera*	2.4	0.9	3.8	0.2	−3.1	3.7	1.3	−1.1	3.7
*Erythrophleum suaveolens*	2.3	1.2	3.4	1.4	−0.6	3.4	0.3	−2.4	3.0
*Nesogordonia kabingaensis*	2.3	1.4	3.2	−0.6	−1.7	0.5	−0.5	−1.5	0.4
*Petersianthus macrocarpus*	3.3	2.3	4.3	−1.5	−3.1	0.1	−1.0	−2.3	0.3
*Pteleopsis hylodendron*	1.5	0.0	3.0	−1.3	−4.0	1.3	−2.0	−5.1	1.2
*Pterocarpus soyauxii*	2.8	1.5	4.1	−1.3	−3.0	0.5	−1.1	−2.8	0.6
*Terminalia superba*	3.9	2.8	4.9	0.0	−1.9	1.8	−1.3	−3.0	0.4
									
Animal dispersed									
*Angylocalyx pynaertii*	2.4	1.3	3.5	−0.2	−1.8	1.4	−0.2	−1.8	1.4
*Celtis adolfi-friderici*	2.9	2.0	3.8	0.0	−1.2	1.2	−0.3	−1.4	0.7
*Celtis mildbraedii*	2.4	2.0	2.9	−0.2	−1.0	0.5	−0.8	−1.4	−0.1
*Cleistopholis patens*	2.4	0.9	4.0	−1.1	−3.3	1.2	−2.0	−4.1	0.1
*Diospyros bipindensis*	0.8	−1.4	2.9	−1.8	−3.5	0.0	−1.8	−3.7	0.1
*Diospyros canaliculata*	0.3	−1.6	2.3	0.1	−1.5	1.8	−1.0	−2.4	0.4
*Greenwayodendron suaveolens*	4.2	3.4	5.0	−0.1	−0.9	0.7	−0.3	−1.0	0.4
*Guarea cedrata*	1.8	0.2	3.3	−0.7	−3.0	1.5	−0.1	−3.3	3.3
*Guarea thompsonii*	2.3	1.3	3.4	−1.1	−2.2	0.1	−1.0	−2.1	0.1
*Lannea welwitschii*	2.1	0.3	3.8	−0.2	−4.5	4.0	0.6	−4.4	5.2
*Macaranga barteri*	2.4	1.1	3.7	−1.0	−2.6	0.6	−0.8	−2.4	0.8
*Staudtia kamerunensis*	1.6	−0.2	3.4	−0.3	−4.1	3.6	0.0	−6.2	6.2
*Strombosia nigropunctata*	1.6	0.6	2.6	−0.5	−1.5	0.5	−0.6	−1.5	0.4
*Strombosia pustulata*	2.2	1.4	3.0	−0.6	−1.5	0.2	−0.3	−1.2	0.6
*Strombosiopsis tetrandra*	3.2	2.2	4.1	−0.9	−2.1	0.3	−0.6	−1.7	0.6
*Xylopia chrysophylla*	1.3	−0.4	3.0	0.1	−3.0	3.2	−2.5	−5.1	0.2
*Xylopia hypolampra*	1.8	0.2	3.4	1.6	−2.0	5.1	0.9	−2.4	4.2
*Xylopia phloiodora*	1.7	0.0	3.3	0.8	−1.4	2.9	1.6	−0.9	4.0
									
Abiotic and animal dispersed									
*Camptostylus mannii*	0.9	−1.1	2.8	0.7	−1.3	2.7	0.3	−1.6	2.1
*Grossera macrantha*	2.3	0.9	3.7	−1.5	−3.0	−0.1	−0.7	−2.2	0.8
*Lepidobotrys staudtii*	1.7	−0.1	3.6	−0.2	−3.0	2.6	0.1	−2.2	2.4
*Myrianthus arboreus*	1.7	0.6	2.7	2.1	−0.3	4.5	2.3	0.7	3.8
*Pausinystalia macroceras*	1.1	0.1	2.1	−0.1	−1.5	1.3	0.3	−1.0	1.6
*Radlkofera calodendron*	2.1	0.0	4.2	−1.9	−4.3	0.7	1.5	−2.4	5.4
*Rinorea oblongifolia*	1.6	−0.5	3.8	1.7	−1.7	5.0	−1.9	−5.2	1.1
*Thomandersia hensii*	2.9	0.0	5.9	1.1	−4.8	6.9	0.0	−6.2	6.2

## Discussion

We find that low-intensity logging affected seed dispersal two decades after the logging event. Guidelines aimed at reducing the ecological damage stemming from logging can substantially reduce short-term impacts (Sist 2000; Sist *et al.* 2003), but our study suggests that impacts of low-intensity logging on ecological processes like seed dispersal are long term and may linger for decades. The difficult-to-detect effects on a key ecological process could have direct consequences for forest species composition through density-dependent recruitment (Janzen 1970; Connell 1971; Cannon *et al.* 1994; Bleher and Böhning-Gaese 2001) and competition at later life stages (Nathan and Muller-Landau 2000), potentially altering the diversity and function of forest ecosystems.

Contrary to our expectations, the dispersal vector of a seed type, abiotic or animal, was not a reliable indicator of the magnitude or direction of the responses of tree species to disturbance. Our results do not support the argument that dispersal decreases for animal-dispersed species following perturbation of the disperser community (Terborgh *et al.* 2008; Markl *et al.* 2012), at least several decades after the fact. It further does not support the notion that dispersal increases for abiotically dispersed species following forest thinning due to increased canopy wind speeds (Gardiner 1994; Stacey *et al.* 1994; Gardiner *et al.* 1997). Our results are more consistent with dispersal effects that are species-specific, as might be expected from the fact that each species has a unique relationship to unmeasured abiotic variables that contribute to its response to disturbance.

Despite a design specifically implemented to detect it, our study did not find evidence for an interaction between hunting and logging for most species, suggesting instead that dispersal following disturbance primarily responds to logging, but not hunting. Using the same data set, Poulsen *et al.* (2013) modelled seed dispersal of nine mammal-dispersed species finding that mean dispersal distance was farther in logged than unlogged forest for five species and farther in unhunted than hunted forest for six species. The disparity between the two studies could be due to the fact that we modelled dispersal for 33 tree species, separating them into animal and abiotic vectors, whereas Poulsen *et al.* (2013) only modelled nine mammal-dispersed species for which they had adequate seed numbers.

Limited evidence for a hunting effect on dispersal could come from the fact that hunting pressures were too low, even where present in our data set. Although hunting has clearly reduced the abundance of large vertebrates in the area (Poulsen *et al.* 2011), all species still exist throughout the landscape (Clark *et al.* 2009)—the vertebrate community is degraded, not defaunated. Alternatively, large frugivorous birds may have replaced the seed dispersal services of large, arboreal mammals. Bird species richness can increase with logging intensity (Burivalova *et al.* 2014), which can aggravate the negative effects of disturbance on seed dispersal due to the reduction in seed dispersers (Moran *et al.* 2004; Kirika *et al.* 2008a, b; Neuschulz *et al.* 2011) or mitigate the effects of disturbance if generalist bird dispersers replace lost or reduced dispersal services (Putz *et al.* 2001; Gray *et al.* 2007; Burivalova *et al.* 2014; LaManna and Martin 2017; Trolliet *et al.* 2017). Indeed, in our study area, there was a 77 % increase in the density of large frugivorous birds following logging (Poulsen *et al.* 2011), a result that is consistent with other sites in the region (Koerner *et al.* 2017). Birds are not commonly hunted in our study site, and 2/3 of the mammal-dispersed species were also dispersed by birds **[see****Supporting Information—Fig. S2****]**, meaning that the full effects of hunting could be attenuated by an expanded bird community.

It is also possible that seed trap data inadequately sample long-distance seed dispersal by animals. A majority of seeds fall locally (Clark *et al.* 1999, 2005; Muller-Landau and Hardesty 2005; Muller-Landau *et al.* 2008), and studies that have combined seed traps with direct observations of seed counts from the canopy (LaDeau and Clark 2001, 2006) or the ground (Minor and Kobe 2017) find seed traps estimate fecundity well. However, seed dispersers may forage over large areas—over 4000 ha in some hornbills (Holbrook and Smith 2000). Seed trap data do not fully capture the dispersal of seeds that are consumed and dispersed outside of the plot. Although long-distance dispersal events may be rare, fully estimating the effects of disturbance on seed dispersal may require combined methods that can account for both local and long-distance dispersal. Nevertheless, our findings indicate that once a forest is disturbed by logging, seed dispersal may be altered regardless of the effect hunting has on seed disperser communities. This is consistent with other studies that found animal guild densities were negatively affected by logging even in the absence of hunting (Poulsen *et al.* 2013), but contradicts studies that found hunting and logging amplified the negative effects of either in isolation (Poulsen *et al.* 2011; Markl *et al.* 2012).

Although dispersal vector was not predictive of how dispersal would respond to hunting or logging, there was a clear distinction in dispersal kernel estimates. Abiotically dispersed seeds moved farthest from the parent tree, animal-dispersed seeds generally fell closest and species dispersed both by animals and abiotically arrived at intermediate distances. Differences in dispersal distance between vectors (Venable and Brown 1988; Greene and Johnson 1989, 1993; Cornelissen *et al.* 2003; Clark *et al.* 2005; Thomson *et al.* 2011) are partly a result of mechanical properties. Abiotically dispersed seeds tend to have small mass that facilitate passive dispersal by wings, plumes, samaras and other adaptations for flight (Greene and Johnson 1989, 1993). Seeds reliant on animal dispersers must develop fleshy fruit mass to entice seed dispersers (Cao *et al.* 2016) limiting their passive dispersal distance.

Estimated fecundity long after disturbance did not differ across disturbance regimes to the extent found in studies immediately following disturbance (Markl *et al.* 2012; Uriarte *et al.* 2012; Berdanier and Clark 2016). Low-intensity logging in resource-limited tropical forest environments may have limited effects on crowding, light and soil moisture levels (Molino and Sabatier 2001; Bongers *et al.* 2009). However, our results suggest that any fecundity benefits from disturbance are unobservable 20 years post-logging. Lack of a long-term effect on fecundity may also be a result of studying only relatively large trees (≥10 cm DBH), which have already made it through the competitive gauntlet of the understory to attain adulthood, and can access resources that facilitate resilience to competitive environments in ways that smaller plants cannot (Clark *et al.* 2004).

Tree size was an important determinant of fecundity making large trees especially important for forest regeneration (Plumptre 1995; Freitas and Pinard 2008). Fecundity of large trees should encourage their protection during logging campaigns (CIB 2006). In addition to their outsized contribution to longer-distance dispersal events (Norghauer *et al.* 2011), large trees store a disproportionate amount of above-ground carbon (Clark and Clark 1996; Lutz *et al.* 2012; Slik *et al.* 2013; Stephenson *et al.* 2014) and are crucial for maintenance of forest structure (Lindenmayer *et al.* 2012; Lutz *et al.* 2013) and animal habitat (Tews *et al.* 2004; Lutz *et al.* 2012, 2013).

Our study demonstrates that disturbances to forests and animal communities contribute to seed dispersal patterns even decades after the initial logging event. In this case, the responses in seed dispersal to disturbance varied across species with weak patterns related to dispersal vector or disturbance type. Our lack of a clear directional effect of hunting and logging on seed dispersal could be partially due to our study design, which was pseudoreplicated: study plots affected by the same disturbance type were geographically grouped together out of necessity. This was a direct result of the study area, particularly the spatial pattern of hunting and logging around the village of Kabo (Poulsen *et al.* 2011), and means that other, unmeasured environmental gradients could influence our results.

The limitations of our study should serve as a challenge to dispersal ecologists and modelers—what are the best methods or combinations of methods for disentangling the effects of multiple disturbances that can operate over disparate spatial and timescales?

Logging concessions cover much of West and Central Africa (FAO 2016), yet the long-term impacts of low-intensity logging techniques on fundamental ecological processes like seed dispersal have been largely overlooked. This work advances our understanding of how the separate and combined effects of hunting and logging affect seed dispersal in the understudied Afrotropics. Although care needs to be taken before extrapolating our results to other contexts, the species-specific dispersal responses to logging in this study point towards the long-lasting toll of disturbance on ecological function. Whereas the effects of disturbance on forest structure and animal communities are easily measured, the effects on ecological processes may be more cryptic, long-lasting and difficult to decipher.

## Data

https://github.com/chasenunez/2018.AOBP.

## Sources of Funding

The U.S. Fish and Wildlife Service Great Ape Fund generously provided financial support for this research. C.L.N. was supported by National Science Foundation (NSF) (GRF-1106401) and a Neil Williams Presidential fellowship; J.R.P. and C.J.C. were supported by a University of Florida Presidential fellowship (J.R.P.), a School of Natural Resources and Environment alumni fellowship (C.J.C.) and Environmental Protection Agency Science to Achieve Results (STAR) fellowships (91630801-0 to J.R.P. and 91643301-0 to C.J.C.); J.S.C. was supported by NSF-EF-1137364, NSF-EF-1550911 and NASA’s AIST programme.

## Contributions by the Authors

C.L.N. posed the central questions, wrote the original manuscript, and analyzed the data; J.S.C. wrote the the R and C++ code for the MASTIF model with testing and feedback by C.L.N. through development; J.R.P. and C.J.C. collected data with help from those in Acknowledgements section; J.R.P. and J.S.C. edited for content and provided guidance on structure and style.

## Conflict of Interest

None declared.

## Supporting Information

The following additional information is available in the online version of this article—

Figure S1. Boxplots comparing the distribution of tree diameters within each plot type show no systematic difference across plot types.

**Figure S2.** Boxplots comparing the distributions of total stems per plot show significant overlap across plot type.

**Figure S3.** Stacked bar plots comparing community composition show a consistent distribution of 33 focal species across plots.

**Figure S4.** Comparison of standardized root mean squared prediction error (individual RMSPE/average number of seeds per trap) with size of circle indicating relative number of seeds from that species present in the study.

**Figure S5.** (A–D) Example of individual results (*Nesogordonia kabingaensis*) that were amalgamated across species for in-text summary figures.

**Figure S6.** (A–C) Examples of model diagnostics for *Nesogordonia kabingaensis.*

**Table S1.** Table of species information and dispersal vectors.

Supplementary Material

## References

[CIT0001] Abernethy KA, Coad L, Taylor G, Lee ME, Maisels F 2013 Extent and ecological consequences of hunting in Central African rainforests in the twenty-first century. Philosophical Transactions of the Royal Society of London. Series B, Biological Sciences 368:20130494. 10.1098/rstb.2012.0303 PMC3720024 23878333

[CIT0002] Beaune D, Bretagnolle F, Bollache L, Hohmann G, Surbeck M, Fruth B 2013 Seed dispersal strategies and the threat of defaunation in a Congo forest. Biodiversity and Conservation 22:225–238.

[CIT0003] Beck H, Snodgrass JW, Thebpanya P 2013 Long-term exclosure of large terrestrial vertebrates: implications of defaunation for seedling demographics in the Amazon rainforest. Biological Conservation 163:115–121.

[CIT0004] Berdanier AB, Clark JS 2015 Multi-year drought-induced morbidity preceding tree death in Southeastern US forests. Ecological Applications 26:150731093536001. 10.1890/15-0274 27039506

[CIT0005] Berdanier AB, Clark JS 2016 Divergent reproductive allocation trade-offs with canopy exposure across tree species in temperate forests. Ecosphere 7:e01313.

[CIT0006] Bleher B, Bohning-Gaese K 2001 Consequences of frugivore diversity for seed dispersal, seedling establishment and the spatial pattern of seedlings and trees. Oecologia 129:385–394. 28547194 10.1007/s004420100747

[CIT0007] Bongers F, Poorter L, Hawthorne WD, Sheil D 2009 The intermediate disturbance hypothesis applies to tropical forests, but disturbance contributes little to tree diversity. Ecology Letters 12:798–805. 19473218 10.1111/j.1461-0248.2009.01329.x

[CIT0008] Boutin S, Wauters LA, McAdam AG, Humphries MM, Tosi G, Dhondt AA 2006 Anticipatory reproduction and population growth in seed predators. Science (New York, N.Y.) 314:1928–1930. 10.1126/science.1135520 17185600

[CIT0009] Brown KA, Gurevitch J 2004 Long-term impacts of logging on forest diversity in Madagascar. Proceedings of the National Academy of Sciences of the United States of America 101:6045–6049. 15067121 10.1073/pnas.0401456101 PMC395920

[CIT0010] Burivalova Z, Sekercioğlu CH, Koh LP 2014 Thresholds of logging intensity to maintain tropical forest biodiversity. Current Biology 24:1893–1898. 25088557 10.1016/j.cub.2014.06.065

[CIT0011] Camargo-Sanabria AA, Mendoza E, Guevara R, Martinez-Ramos M, Dirzo R 2014 Experimental defaunation of terrestrial mammalian herbivores alters tropical rainforest understorey diversity. Proceedings of the Royal Society B: Biological Sciences 282:20142580. 10.1098/rspb.2014.2580 PMC4298212 25540281

[CIT0012] Cannon C, Kartawinata K, Leighton M, Peart David R 1994 The structure of lowland rainforest after selective logging in West Kalimantan, Indonesia. Forest Ecology and Management 67:49–68.

[CIT0013] Cao L, Wang Z, Yan C, Chen J, Guo C, Zhang Z 2016 Differential foraging preferences on seed size by rodents result in higher dispersal success of medium-sized seeds. Ecology 97:3070–3078. 27870042 10.1002/ecy.1555

[CIT0014] Congolaise Industrielle des Bois (CIB) 2006 Plan d’amenagement de l’unité forestière d’aménagement de Kabo (2005–2034). Brazzaville, Republic of the Congo: Ministry of Forest Economy.

[CIT0015] Clark JS 2010 Individuals and the variation needed for high species diversity in forest trees. Science (New York, N.Y.) 327:1129–1132. 10.1126/science.1183506 20185724

[CIT0016] Clark JS, Bell D, Chu C, Courbaud B, Dietze M, Hersh M, HilleRisLambers J, Ibáñez I, LaDeau S, McMahon S, Metcalf J, Mohan J, Moran E, Pangle L, Pearson S, Salk C, Shen Z, Valle D, Wyckoff P 2010 High-dimensional coexistence based on individual variation: a synthesis of evidence. Ecological Monographs 80:569–608.

[CIT0017] Clark JS, Bell DM, Kwit M, Powell A, Zhu K 2013 Dynamic inverse prediction and sensitivity analysis with high-dimensional responses: application to climate-change vulnerability of biodiversity. Journal of Agricultural, Biological, and Environmental Statistics 18:376–404.

[CIT0018] Clark JS, Bell DM, Kwit MC, Zhu K 2014a Competition-interaction landscapes for the joint response of forests to climate change. Global Change Biology 20:1979–1991. 24932467 10.1111/gcb.12425

[CIT0019] Clark DB, Clark DA 1996 Abundance, growth and mortality of very large trees in neotropical lowland rain forest. Forest Ecology and Management 80:235–244.

[CIT0020] Clark JS, Gelfand AE, Woodall CW, Zhu K 2014b More than the sum of the parts: forest climate response from joint species distribution models. Ecological Applications 24:990–999. 25154092 10.1890/13-1015.1

[CIT0021] Clark JS, LaDeau S, Ibanez I 2004 Fecundity of trees and the colonization-competition hypothesis. Ecological Monographs 74:415–442.

[CIT0022] Clark JS, Macklin E, Wood L 1998 Stages and spatial scales of recruitment limitation in southern Appalachian forests. Ecological Monographs 68:213–235.

[CIT0023] Clark CJ, Poulsen JR, Bolker BM, Connor EF, Parker VT 2005 Comparative seed shadows of bird-, monkey-, and wind-dispersed trees. Ecology 86:2684–2694.

[CIT0024] Clark CJ, Poulsen JR, Malonga R, Elkan PW 2009 Logging concessions can extend the conservation estate for central African tropical forests. Conservation Biology 23:1281–1293. 19453655 10.1111/j.1523-1739.2009.01243.x

[CIT0025] Clark CJ, Poulsen JR, Parker VT 2001 The role of arboreal seed dispersal groups on the seed rain of a lowland tropical forest. Biotropica 33:606–620.

[CIT0026] Clark JS, Silman M, Kern R, Macklin E, Hillerislambers J 1999 Seed dispersal near and far: patterns across temperate and tropical forests. Ecology 80:1475–1494.

[CIT0027] Comita LS, Queenborough SA, Murphy SJ, Eck JL, Xu K, Krishnadas M, Beckman N, Zhu Y, Gomez-Aparicio L 2014 Testing predictions of the Janzen-Connell hypothesis: a metaanalysis of experimental evidence for distance- and density dependent seed and seedling survival. The Journal of Ecology 102:845–856. 25253908 10.1111/1365-2745.12232 PMC4140603

[CIT0028] Connell JH 1971 On the role of natural enemies in preventing competitive exclusion in some marine animals and in rain forest trees. In: Den Boer PJ, Gradwell GR, eds. Dynamics of populations. Wageningen, The Netherlands: Centre for Agricultural Publishing and Documentation, 298–312.

[CIT0029] Cornelissen JHC, Lavorel S, Garnier E, Díaz S, Buchmann N, Gurvich DE, Reich PB, ter Steege H, Morgan HD, van der Heijden MGA, Pausas JG, Poorter H 2003 A handbook of protocols for standardised and easy measurement of plant functional traits worldwide. Australian Journal of Botany 51:335–380.

[CIT0030] Dirzo R, Young HS, Galetti M, Ceballos G, Isaac NJ, Collen B 2014 Defaunation in the Anthropocene. Science (New York, N.Y.) 345:401–406. 10.1126/science.1251817 25061202

[CIT0031] Edwards DP, Tobias JA, Sheil D, Meijaard E, Laurance WF 2014 Maintaining ecosystem function and services in logged tropical forests. Trends in Ecology & Evolution 29:511–520. 25092495 10.1016/j.tree.2014.07.003

[CIT0032] Ewel JJ, Mazzarino MJ 2008 Competition from below for light and nutrients shifts productivity among tropical species. Proceedings of the National Academy of Sciences of the United States of America 105:18836–18841. 19022907 10.1073/pnas.0807216105 PMC2596203

[CIT0033] FAO 2016 The contemporary forest concessions in West and Central Africa: chronicle of a foretold decline? Forestry Policy and Institutions Working Paper 34. Rome: FAO.

[CIT0034] Freitas JV de, Pinard MA 2008 Applying ecological knowledge to decisions about seed tree retention in selective logging in tropical forests. Forest Ecology and Management 256:1434–1442.

[CIT0035] Gardiner BA 1994 Wind and wind forces in a plantation spruce forest. Boundary-Layer Meteorology 67:161–186.

[CIT0036] Gardiner BA, Stagey GR, Belcher RE, Wood CJ 1997 Field and wind tunnel assessments of the implications of respacing and thinning for tree stability. Forestry 70:233–252.

[CIT0037] Gautier-Hion A, Duplantier J-M, Quris R, Feer F, Sourd C, Decoux JP, Dubost G, Emmons L, Erard C, Hecketsweiler P, Moungazi A, Roussilhon C, Thiollay J-M 1985 Fruit characters as a basis of fruit choice and seed dispersal in a tropical forest vertebrate community. Oecologia 65:324–337. 28310436 10.1007/BF00378906

[CIT0038] Gourlet-Fleury S, Mortier F, Fayolle A, Baya F, Ouedraogo D, Benedet F, Picard N 2013 Tropical forest recovery from logging: a 24 year silvicultural experiment from Central Africa. Philosophical Transactions of the Royal Society of London. Series B, Biological Sciences 368:20120302. 23878332 10.1098/rstb.2012.0302 PMC3720023

[CIT0039] Gray MA, Baldauf SL, Mayhew PJ, Hill JK 2007 The response of avian feeding guilds to tropical forest disturbance. Conservation Biology 21:133–141. 17298519 10.1111/j.1523-1739.2006.00557.x

[CIT0040] Greene DF, Johnson EA 1989 A model of wind dispersal of winged or plumed seeds. Ecology 70:339–347.

[CIT0041] Greene D, Johnson E 1993 Seed mass and dispersal capacity in wind-dispersed diaspores. Oikos 67:69–74.

[CIT0042] Gutierrez-Granados G 2011 Effect of logging on rodent scatterhoarding dynamics in tropical forests: implications for plant recruitment. Integrative Zoology 6:74–80. 21645273 10.1111/j.1749-4877.2011.00234.x

[CIT0043] Haurez B, Tagg N, Petre C-A, Vermeulen C, Doucet J-L 2016 Short term impact of selective logging on a western lowland gorilla population. Forest Ecology and Management 364:46–51.

[CIT0044] Hawthorne W, Gyakari N 2006 Photoguide for the forest trees of Ghana: a tree-spotter’s field guide for identifying the largest trees. Oxford: Oxford Forestry Institute.

[CIT0045] Hoffmann M, Hilton-Taylor C, Angulo A, Böhm M, Brooks TM, Butchart SH, Carpenter KE, Chanson J, Collen B, Cox NA, Darwall WR, Dulvy NK, Harrison LR, Katariya V, Pollock CM, Quader S, Richman NI, Rodrigues AS, Tognelli MF, Vié JC, Aguiar JM, Allen DJ, Allen GR, Amori G, Ananjeva NB, Andreone F, Andrew P, Aquino Ortiz AL, Baillie JE, Baldi R, Bell BD, Biju SD, Bird JP, Black-Decima P, Blanc JJ, Bolaños F, Bolivar-G W, Burfield IJ, Burton JA, Capper DR, Castro F, Catullo G, Cavanagh RD, Channing A, Chao NL, Chenery AM, Chiozza F, Clausnitzer V, Collar NJ, Collett LC, Collette BB, Cortez Fernandez CF, Craig MT, Crosby MJ, Cumberlidge N, Cuttelod A, Derocher AE, Diesmos AC, Donaldson JS, Duckworth JW, Dutson G, Dutta SK, Emslie RH, Farjon A, Fowler S, Freyhof J, Garshelis DL, Gerlach J, Gower DJ, Grant TD, Hammerson GA, Harris RB, Heaney LR, Hedges SB, Hero JM, Hughes B, Hussain SA, Icochea MJ, Inger RF, Ishii N, Iskandar DT, Jenkins RK, Kaneko Y, Kottelat M, Kovacs KM, Kuzmin SL, La Marca E, Lamoreux JF, Lau MW, Lavilla EO, Leus K, Lewison RL, Lichtenstein G, Livingstone SR, Lukoschek V, Mallon DP, McGowan PJ, McIvor A, Moehlman PD, Molur S, Muñoz Alonso A, Musick JA, Nowell K, Nussbaum RA, Olech W, Orlov NL, Papenfuss TJ, Parra-Olea G, Perrin WF, Polidoro BA, Pourkazemi M, Racey PA, Ragle JS, Ram M, Rathbun G, Reynolds RP, Rhodin AG, Richards SJ, Rodríguez LO, Ron SR, Rondinini C, Rylands AB, Sadovy de Mitcheson Y, Sanciangco JC, Sanders KL, Santos-Barrera G, Schipper J, Self-Sullivan C, Shi Y, Shoemaker A, Short FT, Sillero-Zubiri C, Silvano DL, Smith KG, Smith AT, Snoeks J, Stattersfield AJ, Symes AJ, Taber AB, Talukdar BK, Temple HJ, Timmins R, Tobias JA, Tsytsulina K, Tweddle D, Ubeda C, Valenti SV, van Dijk PP, Veiga LM, Veloso A, Wege DC, Wilkinson M, Williamson EA, Xie F, Young BE, Akçakaya HR, Bennun L, Blackburn TM, Boitani L, Dublin HT, da Fonseca GA, Gascon C, Lacher TE Jr, Mace GM, Mainka SA, McNeely JA, Mittermeier RA, Reid GM, Rodriguez JP, Rosenberg AA, Samways MJ, Smart J, Stein BA, Stuart SN 2010 The impact of conservation on the status of the world’s vertebrates. Science 330:1503–1509. 20978281 10.1126/science.1194442

[CIT0046] Holbrook KM, Smith TB 2000 Seed dispersal and movement patterns in two species of *Ceratogymna* hornbills in a West African tropical lowland forest. Oecologia 125:249–257. 24595836 10.1007/s004420000445

[CIT0047] Huante P, Rincon E, Chapin Iii FS 1998 Foraging for nutrients, responses to changes in light, and competition in tropical deciduous tree seedlings. Oecologia 117:209–216. 28308489 10.1007/s004420050650

[CIT0048] Janzen DH 1970 Herbivores and the number of tree species in tropical forests. The American Naturalist 104:501–528.

[CIT0049] John R, Dalling JW, Harms KE, Yavitt JB, Stallard RF, Mirabello M, Hubbell SP, Valencia R, Navarrete H, Vallejo M, Foster RB 2007 Soil nutrients influence spatial distributions of tropical tree species. Proceedings of the National Academy of Sciences of the United States of America 104:864–869. 17215353 10.1073/pnas.0604666104 PMC1783405

[CIT0050] Johns AD 1988 Effects of “selective” timber extraction on rain forest structure and composition and some consequences for frugivores and folivores. Source: Biotropica 20:31–37.

[CIT0051] Kasenene JM, Murphy PG 1991 Post-logging tree mortality and major branch losses in Kibale Forest, Uganda. Forest Ecology and Management 46:295–307.

[CIT0052] Kirika JM, Bleher B, Bohning-Gaese K, Chira R, Farwig N 2008a Fragmentation and local disturbance of forests reduce frugivore diversity and fruit removal in *Ficus thonningii* trees. Basic and Applied Ecology 9:663–672.

[CIT0053] Kirika JM, Farwig N, Bohning-Gaese K 2008b Effects of local disturbance of tropical forests on frugivores and seed removal of a small-seeded Afrotropical tree. Conservation Biology 22:318–328. 18241235 10.1111/j.1523-1739.2007.00874.x

[CIT0054] Kleinschroth F, Healey JR 2017 Impacts of logging roads on tropical forests. Biotropica 49:620–635.

[CIT0055] Koenig WD, Knops JMH 2001 Seed-crop size and eruptions of North American boreal seed-eating birds. Journal of Animal Ecology 70:609–620.

[CIT0056] Koerner SE, Poulsen JR, Blanchard EJ, Okouyi J, Clark CJ 2017 Vertebrate community composition and diversity declines along a defaunation gradient radiating from rural villages in Gabon. Journal of Applied Ecology 54:805–814.

[CIT0057] Kurten EL 2013 Cascading effects of contemporaneous defaunation on tropical forest communities. Biological Conservation 163:22–32.

[CIT0058] LaDeau SL, Clark JS 2001 Rising CO_2_ levels and the fecundity of forest trees. Science 292:95–98. 11292871 10.1126/science.1057547

[CIT0059] LaDeau SL, Clark JS 2006 Elevated CO_2_ and tree fecundity: the role of tree size, interannual variability, and population heterogeneity. Global Change Biology 12:822–833.

[CIT0060] LaManna JA, Martin TE 2017 Logging impacts on avian species richness and composition differ across latitudes and foraging and breeding habitat preferences. Biological Reviews of the Cambridge Philosophical Society 92:1657–1674. 27723232 10.1111/brv.12300

[CIT0061] Laporte NT, Stabach JA, Grosch R, Lin TS, Goetz SJ 2007 Expansion of industrial logging in Central Africa. Science (New York, N.Y.) 316:1451. 10.1126/science.1141057 17556578

[CIT0062] Laurance WF, Delamonica P, Laurance SG, Vasconcelos HL, Lovejoy TE 2000 Rainforest fragmentation kills big trees. Nature 404:836. 10786782 10.1038/35009032

[CIT0063] Lewis SL, Phillips OL, Sheil D, Vinceti B, Baker TR, Brown S, Graham AW, Higuchi N, Hilbert DW, Laurance WF, Lejoly J, Malhi Y, Monteagudo A, Vargas PN, Sonké B, Supardi MNN, Terborgh JW, Martínez RV 2004 Tropical forest tree mortality, recruitment and turnover rates: calculation, interpretation and comparison when census intervals vary. Journal of Ecology 92:929–944.

[CIT0064] Lindenmayer DB, Laurance WF, Franklin JF 2012 Ecology. Global decline in large old trees. Science (New York, N.Y.) 338:1305–1306. 10.1126/science.1231070 23224548

[CIT0065] Lutz JA, Larson AJ, Freund JA, Swanson ME, Bible KJ 2013 The importance of large-diameter trees to forest structural heterogeneity. PLoS One 8:e82784. 24376579 10.1371/journal.pone.0082784 PMC3869720

[CIT0066] Lutz JA, Larson AJ, Swanson ME, Freund JA 2012 Ecological importance of large-diameter trees in a temperate mixed-conifer forest. PLoS One 7:e36131. 22567132 10.1371/journal.pone.0036131 PMC3342248

[CIT0067] Markl JS, Schleuning M, Forget PM, Jordano P, Lambert JE, Traveset A, Wright SJ, Bohning-Gaese K 2012 Meta-analysis of the effects of human disturbance on seed dispersal by animals. Conservation Biology 26:1072–1081. 22971077 10.1111/j.1523-1739.2012.01927.x

[CIT0068] Martinez I, Gonzalez-Taboada F 2009 Seed dispersal patterns in a temperate forest during a mast event: performance of alternative dispersal kernels. Oecologia 159:389–400. 19018573 10.1007/s00442-008-1218-4

[CIT0069] Medjibe VP, Putz FE, Romero C 2013 Certified and uncertified logging concessions compared in Gabon: changes in stand structure, tree species, and biomass. Environmental Management 51:524–540. 23277438 10.1007/s00267-012-0006-4

[CIT0070] Meijaard E, Sheil D, Nasi R, Augeri D, Rosenbaum B, Iskandar D, Setyawati T, Lammertink M, Rachmatika I, Wong A, Soehartono T, Stanley S, O’Brien T 2005 Life after logging. Reconciling wildlife conservation and production forestry in Indonesian Borneo. Bogor, Indonesia: Center for International Forestry Research (CIFOR).

[CIT0071] Minor DM, Kobe RK 2017 Masting synchrony in northern hardwood forests: super-producers govern population fruit production. Journal of Ecology 105:987–998.

[CIT0072] Mockrin MH 2008 The spatial structure and sustainability of subsistence wildlife harvesting in Kabo, Congo. PhD Thesis, Columbia University of Utrecht, USA.

[CIT0073] Molino JF, Sabatier D 2001 Tree diversity in tropical rain forests: a validation of the intermediate disturbance hypothesis. Science (New York, N.Y.) 294:1702–1704. 10.1126/science.1060284 11721052

[CIT0074] Moran C, Catterall CP, Green RJ, Olsen MF 2004 Functional variation among frugivorous birds: implications for rainforest seed dispersal in a fragmented subtropical landscape. Oecologia 141:584–595. 15309614 10.1007/s00442-004-1685-1

[CIT0075] Morgan D, Sanz C 2006 Chimpanzee feeding ecology and comparisons with sympatric gorillas in the Goualougo Triangle, Republic of Congo. In: Hohmann G, Robbins M, Boesch C, eds. Feeding ecology in apes and other primates. Cambridge: Cambridge University Press, 97–122.

[CIT0076] Muller-Landau HC, Hardesty BD 2005 Seed dispersal of woody plants in tropical forests : concepts, examples and future directions. In: Burslem DFRP, Pinard MA, Hartley SE, eds. Biotic interactions in the tropic. Cambridge, UK: Cambridge University Press, 267–309.

[CIT0077] Muller-Landau HC, Wright SJ, Calderon O, Condit R, Hubbell SP 2008 Interspecific variation in primary seed dispersal in a tropical forest. Journal of Ecology 96:653–667.

[CIT0078] Nathan R, Muller-Landau HC 2000 Spatial patterns of seed dispersal, their determinants and consequences for recruitment. Trends in Ecology & Evolution 15:278–285. 10856948 10.1016/s0169-5347(00)01874-7

[CIT0079] Neuschulz EL, Botzat A, Farwig N 2011 Effects of forest modification on bird community composition and seed removal in a heterogeneous landscape in South Africa. Oikos 120:1371–1379.

[CIT0080] Norghauer JM, Nock CA, Grogan J 2011 The importance of tree size and fecundity for wind dispersal of big-leaf mahogany. PLoS One 6:e17488. 21408184 10.1371/journal.pone.0017488 PMC3049789

[CIT0081] Pinard MA, Barker MG, Tay J 2000 Soil disturbance and postlogging forest recovery on bulldozer paths in Sabah, Malaysia. Forest Ecology and Management 130:213–225.

[CIT0082] Plumptre AJ 1995 The importance of “seed trees” for the natural regeneration of selectively logged tropical forest. Commonwealth Forestry Review 74:253–258.

[CIT0083] Poulsen JR, Clark CJ, Bolker BM 2011 Decoupling the effects of logging and hunting on an Afrotropical animal community. Ecological Applications 21:1819–1836. 21830721 10.1890/10-1083.1

[CIT0084] Poulsen JR, Clark CJ, Connor EF, Smith TB 2002 Differential resource use by primates and hornbills: implications for seed dispersal. Ecology 83:228–240.

[CIT0085] Poulsen JR, Clark CJ, Mavah G, Elkan PW 2009 Bushmeat supply and consumption in a tropical logging concession in northern Congo. Conservation Biology 23:1597–1608. 19459888 10.1111/j.1523-1739.2009.01251.x

[CIT0086] Poulsen JR, Clark CJ, Palmer TM 2013 Ecological erosion of an Afrotropical forest and potential consequences for tree recruitment and forest biomass. Biological Conservation 163:122–130.

[CIT0087] Poulsen JR, Clark CJ, Smith TB 2001 Seed dispersal by a diurnal primate community in the Dja Reserve, Cameroon. Journal of Tropical Ecology 17:787–808.

[CIT0088] Putz FE, Blate GM, Redford KH, Fimbel R, Robinson J 2001 Tropical forest management and conservation of biodiversity: an overview. Conservation Biology 15:7–20.

[CIT0089] Rosin C, Poulsen JR 2016 Hunting-induced defaunation drives increased seed predation and decreased seedling establishment of commercially important tree species in an Afrotropical forest. Forest Ecology and Management 382:206–213.

[CIT0090] Schnitzer SA, Bongers F 2002 The ecology of lianas and their role in forests. Trends in Ecology and Evolution 17:223–230.

[CIT0091] Sist P 2000 Reduced-impact logging in the tropics: objectives, principles and impacts. International Forestry Review 2:3–10.

[CIT0092] Sist P, Fimbel R, Sheil D, Nasi R, Chevallier MH 2003 Towards sustainable management of mixed dipterocarp forests of Southeast Asia: moving beyond minimum diameter cutting limits. Environmental Conservation 30:364–374.

[CIT0093] Slik JWF, Paoli G, Mcguire K, Amaral I, Barroso J, Bastian M, Blanc L, Bongers F, Boundja P, Clark C, Collins M, Dauby G, Ding Y, Doucet JL, Eler E, Ferreira L, Forshed O, Fredriksson G, Gillet JF, Harris D, Leal M, Laumonier Y, Malhi Y, Mansor A, Martin E, Miyamoto K, Araujo-Murakami A, Nagamasu H, Nilus R, Nurtjahya E, Oliveira A, Onrizal O, Parada-Gutierrez A, Permana A, Poorter L, Poulsen J, Ramirez-Angulo H, Reitsma J, Rovero F, Rozak A, Sheil D, Silva-Espejo J, Silveira M, Spironelo W, ter Steege H, Stevart T, Navarro-Aguilar GE, Sunderland T, Suzuki E, Tang J, Theilade I, van der Heijden G, van Valkenburg J, VanDo T, Vilanova E, Vos V, Wich S, Wöll H, Yoneda T, Zang R, Zhang MG, Zweifel N 2013 Large trees drive forest aboveground biomass variation in moist lowland forests across the tropics. Global Ecology and Biogeography 22:1261–1271.

[CIT0094] Stacey GR, Belcher RE, Wood CJ, Gardiner BA 1994 Wind flows and forces in a model spruce forest. Boundary-Layer Meteorology 69:311–334.

[CIT0095] Stephenson NL, Das AJ, Condit R, Russo SE, Baker PJ, Beckman NG, Coomes DA, Lines ER, Morris WK, Ruger N, Alvarez E, Blundo C, Bunyavejchewin S, Chuyong G, Davies SJ, Duque A, Ewango CN, Flores O, Franklin JF, Grau HR, Hao Z, Harmon ME, Hubbell SP, Kenfack D, Lin Y, Makana JR, Malizia A, Malizia LR, Pabst RJ, Pongpattananurak N, Su SH, Sun IF, Tan S, Thomas D, van Mantgem PJ, Wang X, Wiser SK, Zavala MA 2014 Rate of tree carbon accumulation increases continuously with tree size. Nature 507:90–93. 24429523 10.1038/nature12914

[CIT0096] Terborgh J, Nunez-Iturri G, Pitman NC, Valverde FH, Alvarez P, Swamy V, Pringle EG, Paine CE 2008 Tree recruitment in an empty forest. Ecology 89:1757–1768. 18589539 10.1890/07-0479.1

[CIT0097] Tews J, Brose U, Grimm V, Tielbörger K, Wichmann MC, Schwager M, Jeltsch F 2004 Animal species diversity driven by habitat heterogeneity/diversity: the importance of keystone structures. Journal of Biogeography 31:79–92.

[CIT0098] Theimer TC, Gehring CA, Green PT, Connell JH 2011 Terrestrial vertebrates alter seedling composition and richness but not diversity in an Australian tropical rain forest. Ecology 92:1637–1647. 21905430 10.1890/10-2231.1

[CIT0099] Thomson FJ, Moles AT, Auld TD, Kingsford RT 2011 Seed dispersal distance is more strongly correlated with plant height than with seed mass. Journal of Ecology 99:1299–1307.

[CIT0100] Trolliet F, Forget PM, Doucet JL, Gillet JF, Hambuckers A 2017 Frugivorous birds influence the spatial organization of tropical forests through the generation of seedling recruitment foci under zoochoric trees. Acta Oecologica 85:69–76.

[CIT0101] Tutin CE, Ham RM, White LJ, Harrison MJ 1997 The primate community of the Lope Reserve, Gabon: diets, responses to fruit scarcity, and effects on biomass. American Journal of Primatology 42:1–24. 9108968 10.1002/(SICI)1098-2345(1997)42:1<1::AID-AJP1>3.0.CO;2-0

[CIT0102] Uriarte M, Clark JS, Zimmerman JK, Comita LS, Forero-Montana J, Thompson J 2012 Multidimensional trade-offs in species responses to disturbance: implications for diversity in a subtropical forest. Ecology 93:191–205. 22486099 10.1890/10-2422.1

[CIT0103] Venable DL, Brown JS 1988 The selective interactions of dispersal, dormancy, and seed size as adaptations for reducing risk in variable environments. The American Naturalist 131:360–384.

[CIT0104] Wang Y, Zhang J, LaMontagne JM, Lin F, Li B, Ye J, Yuan Z, Wang X, Hao Z 2017 Variation and synchrony of tree species mast seeding in an old-growth temperate forest. Journal of Vegetation Science 28:413–423.

[CIT0105] White LJT, Abernethy K 1997 A guide to the vegetation of the Lopé Reserve, Gabon. 2nd edn Libreville: Multipress-Gabon.

[CIT0106] Whitney KD, Fogiel MK, Lamperti AM, Holbrook KM, Stauffer DJ, Hardesty BD, Thomas Parker V, Smith TB 1998 Seed dispersal by *Ceratogymna* hornbills in the Dja Reserve, Cameroon. Journal of Tropical Ecology 14:351–371.

[CIT0107] Willson M, Traveset A 2000 The ecology of seed dispersal. In: Fenner M, ed. Seeds: the ecology of regeneration in plant communities. Oxfordshire, UK: Centre for Agriculture and Bioscience International, 85–110.

[CIT0108] Wortley AH, Harris DJ 2014 Sangha trees: an identification and training guide to the trees of the northern republic of Congo. In: Watson MF, Lyal CHC, Pendry CA, eds. Descriptive taxonomy: the foundation of biodiversity research. Cambridge, UK: Cambridge University Press, 127–145.

