# Supplementary material for: Low-intensity logging and hunting have long-term effects on seed dispersal but not fecundity in Afrotropical forests
Source: AoB Plants. 2018 Dec 13;11(1):ply074. doi: 10.1093/aobpla/ply074 (PMC6346634; doi:10.1093/aobpla/ply074)
Supplement: Supplementary Material [file ply074_suppl_supplementary_material.pdf]

SUPPLEMENT

Figure S1: Boxplots comparing the distribution of tree diameters within each plot type show no systematic difference across plot types.

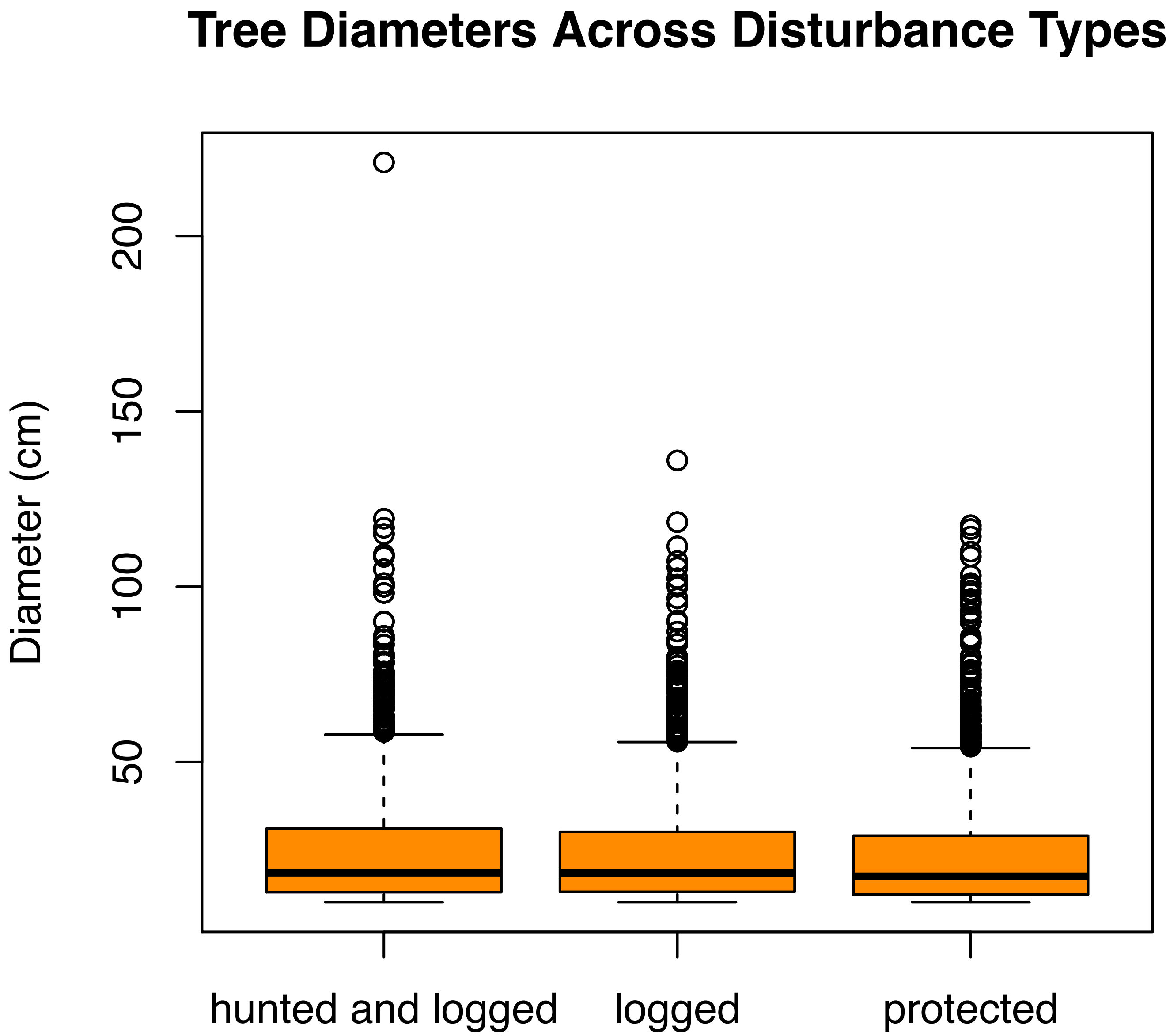

Figure S2: Boxplots comparing the distributions of total stems per plot show significant overlap across plot type.

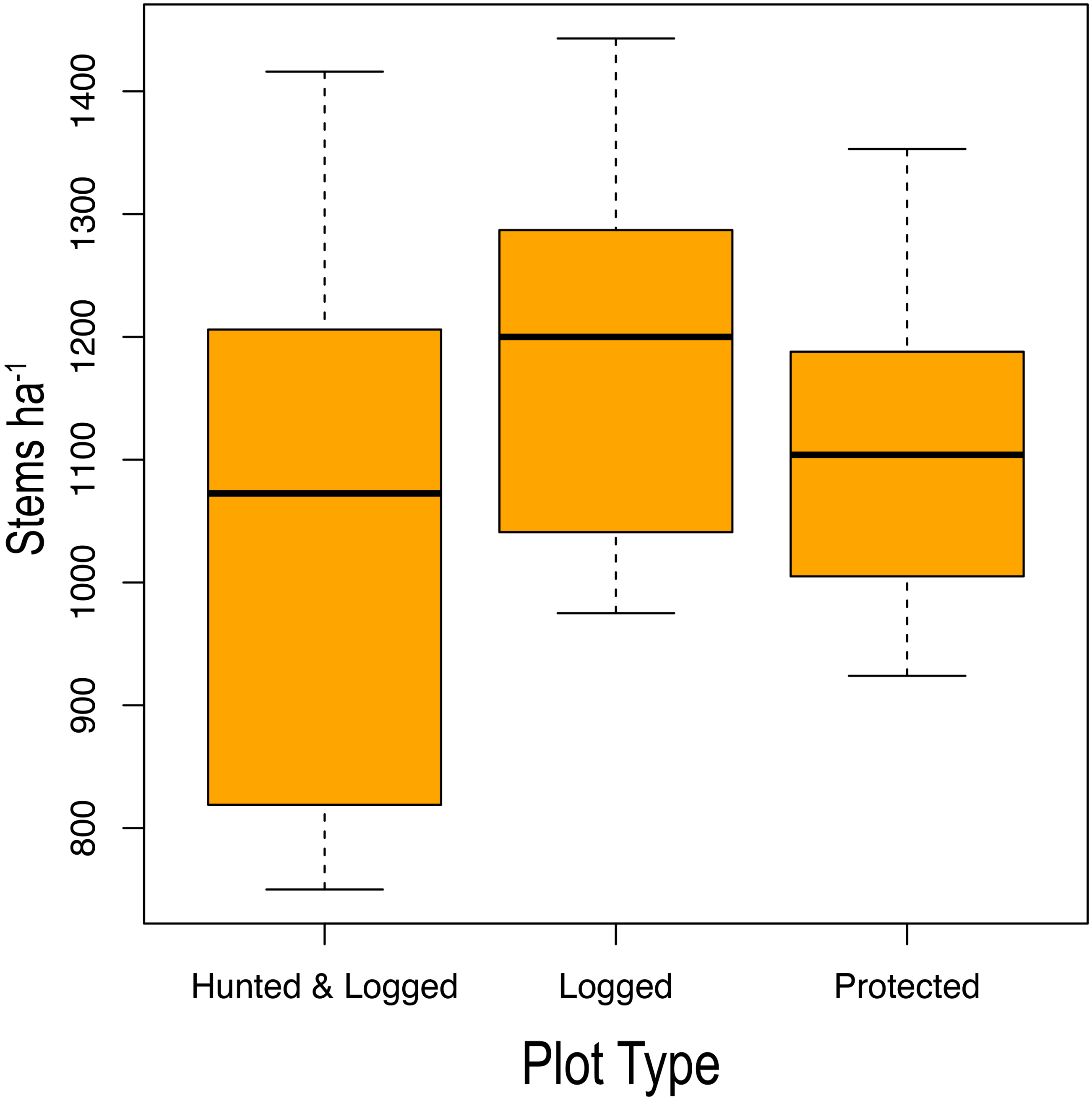

Figure S3: Stacked bar plots comparing community composition show a consistent distribution of 33 focal species across plots.

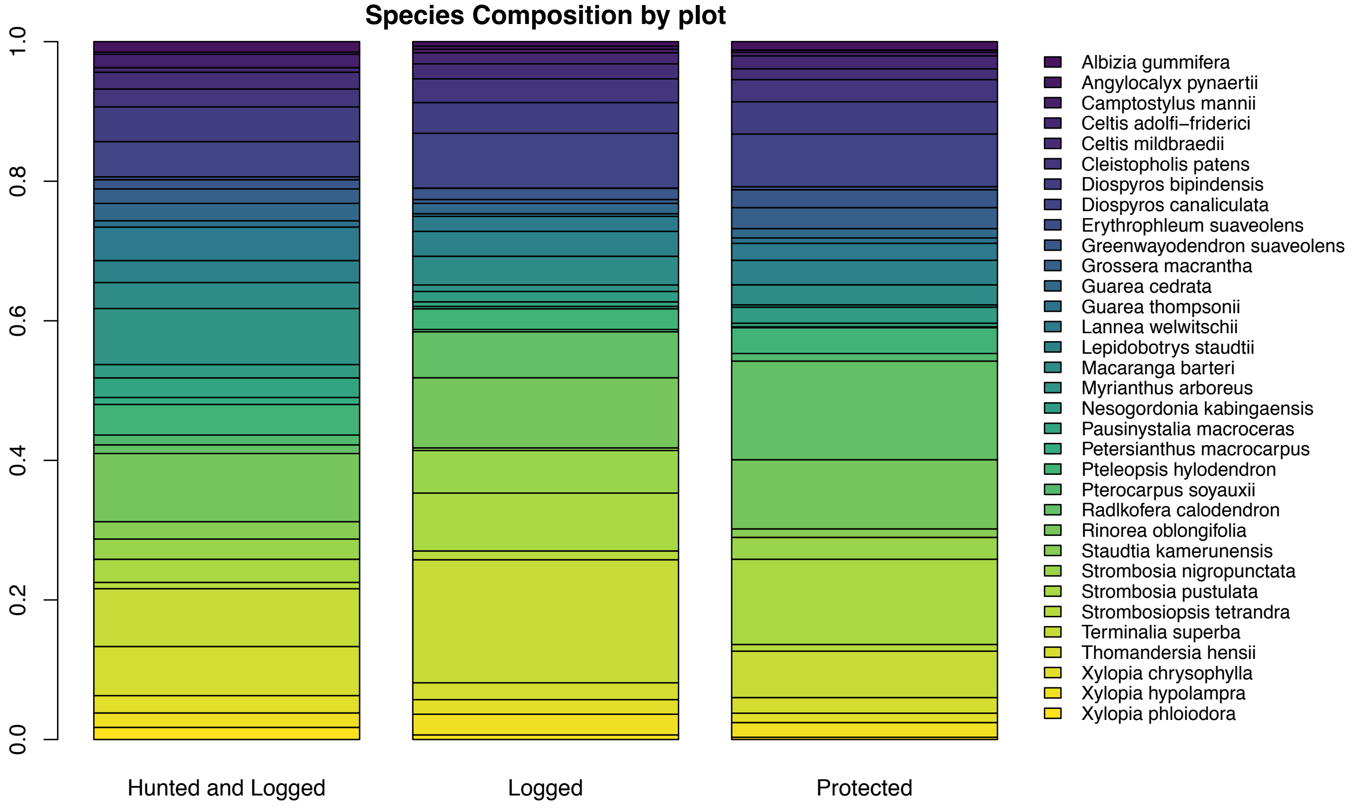

Figure S4: Comparison of standardized root mean squared prediction error (individual RMSPE/average number of seeds per trap) with size of circle indicating relative number of seeds from that species present in the study.

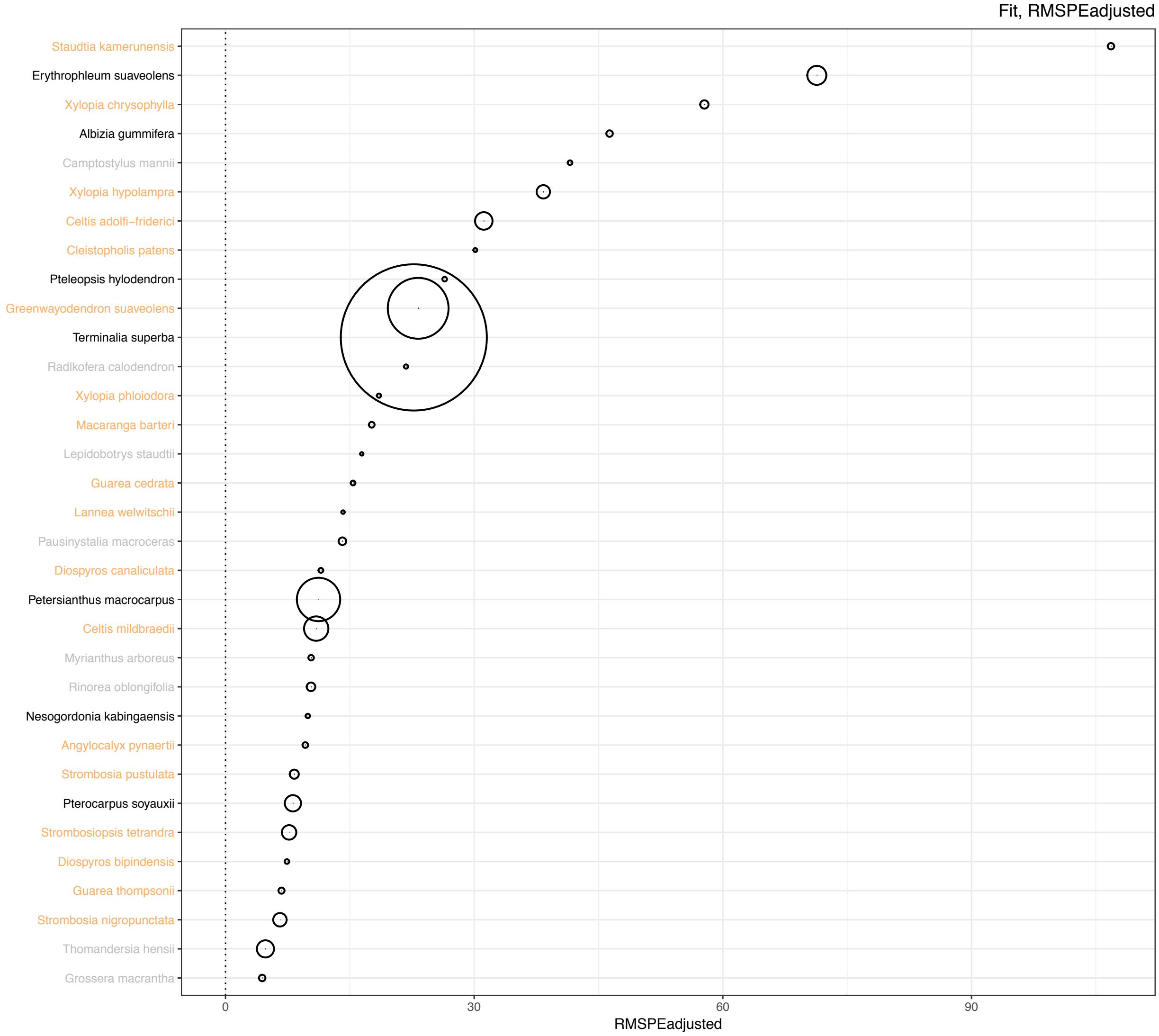

Figure S5 (A-D): Example of individual results (*Nesogordonia kabingaensis)* that were amalgamated across species for in-text summary figures.

S5A: comparison of the dispersal parameter and kernel mean dispersal distance among plot types.

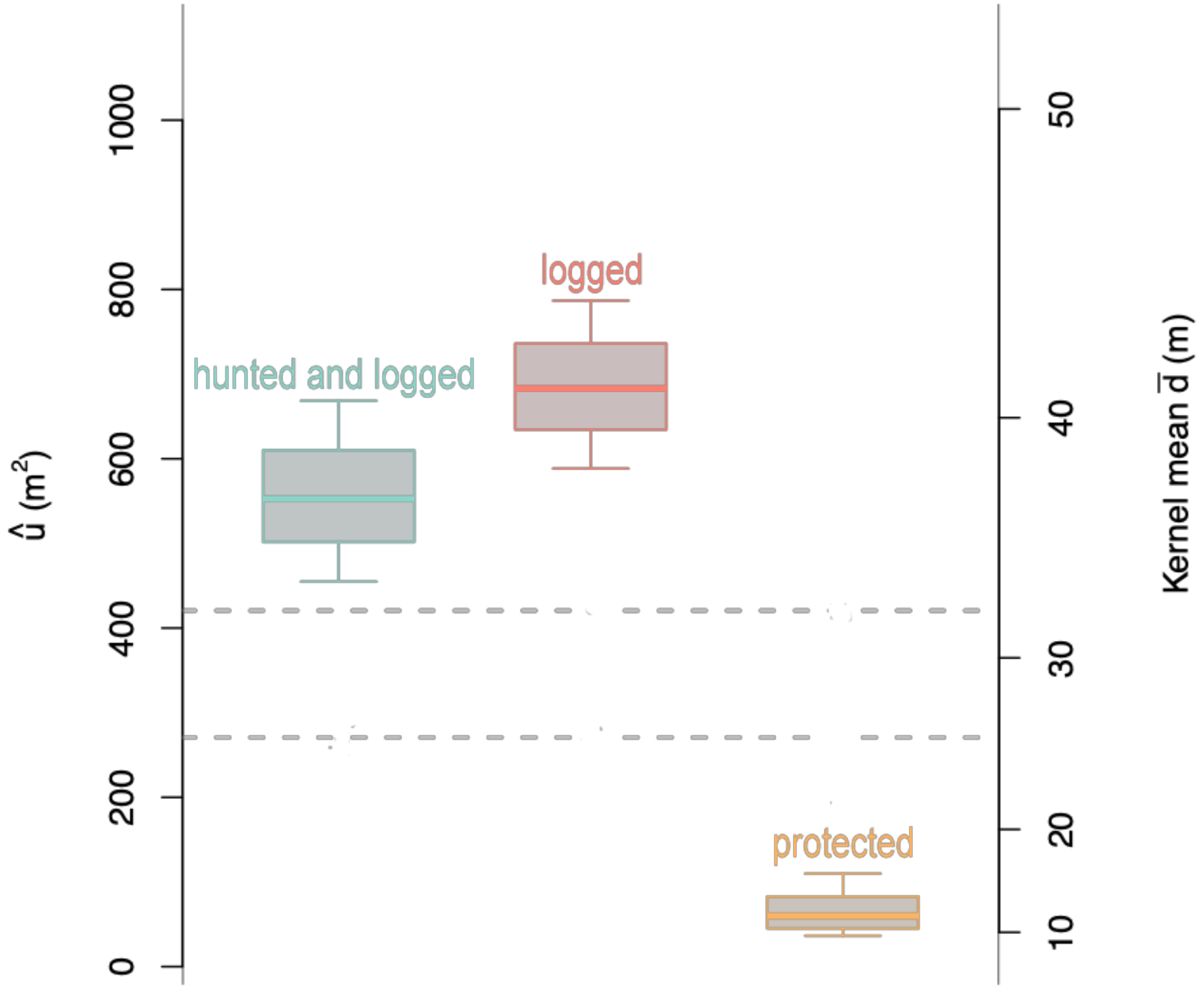

S5B: Posterior parameter estimates of maturation and fecundity for *Nesogordonia kabingaensis.*

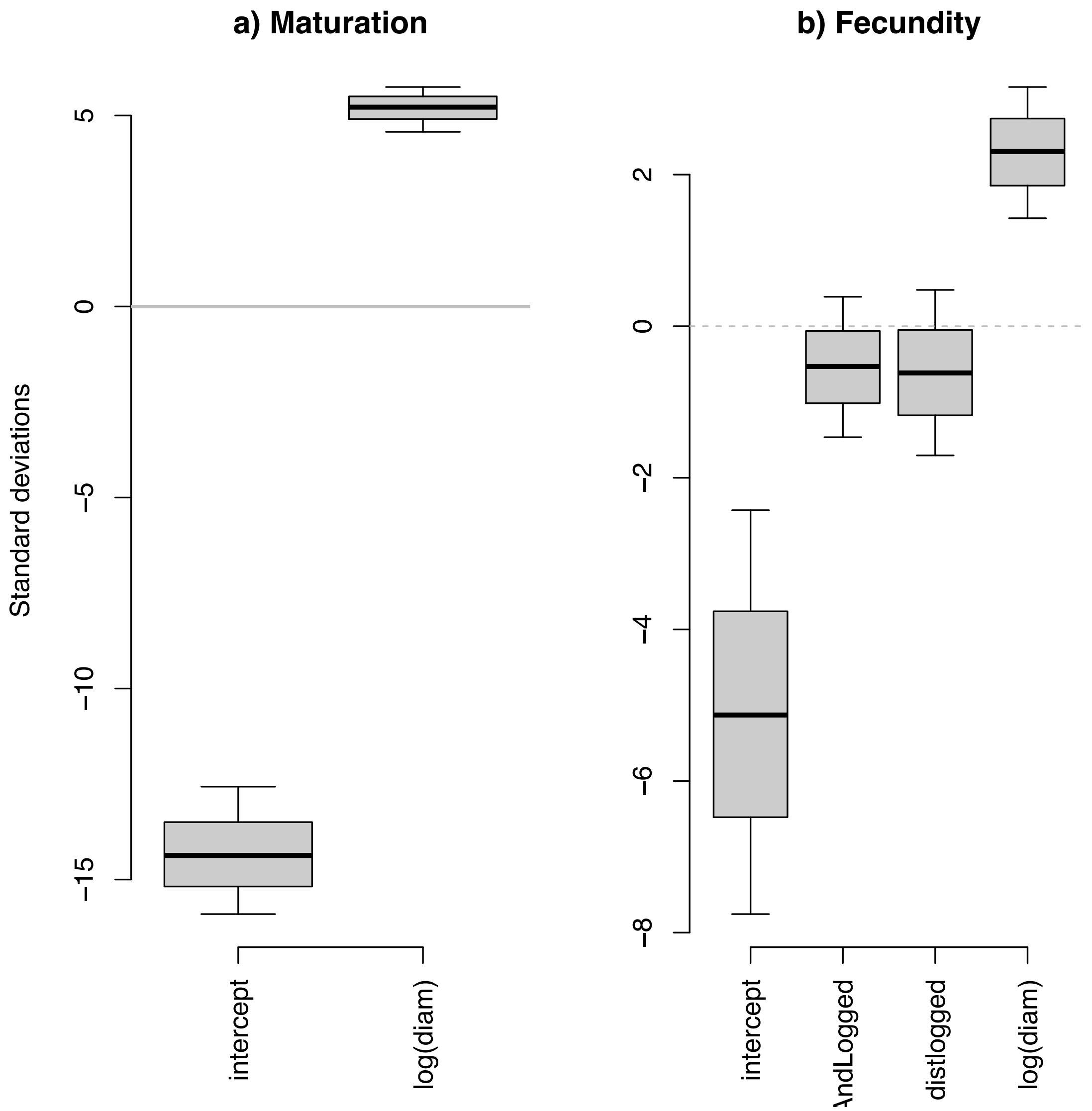

S5C: Posterior estimate of maturity as a function of tree diameter for *Nesogordonia kabingaensis.*

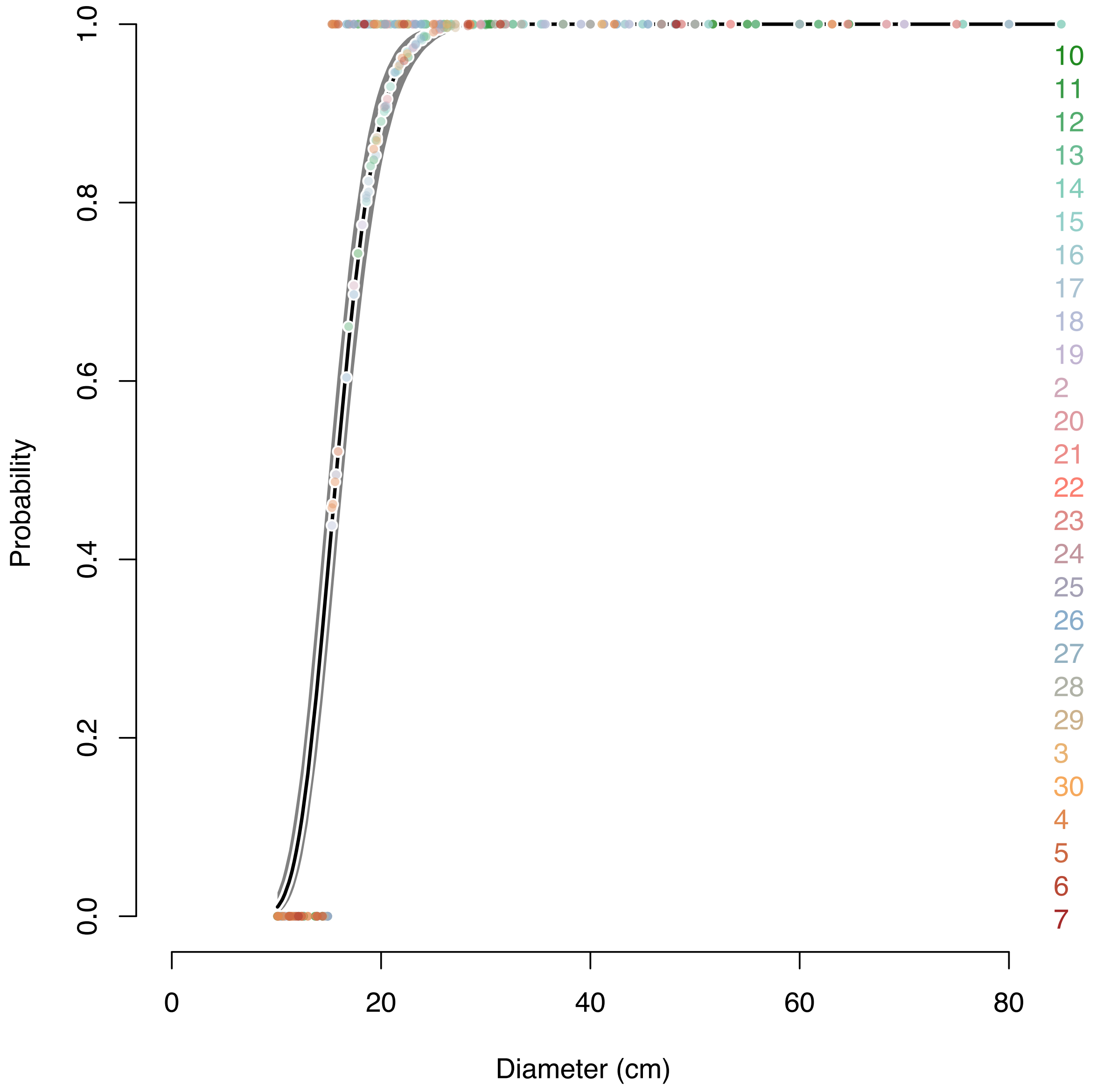

S5D: Comparison of seed dispersal kernels with 95% CI across plot types for *Nesogordonia kabingaensis.*

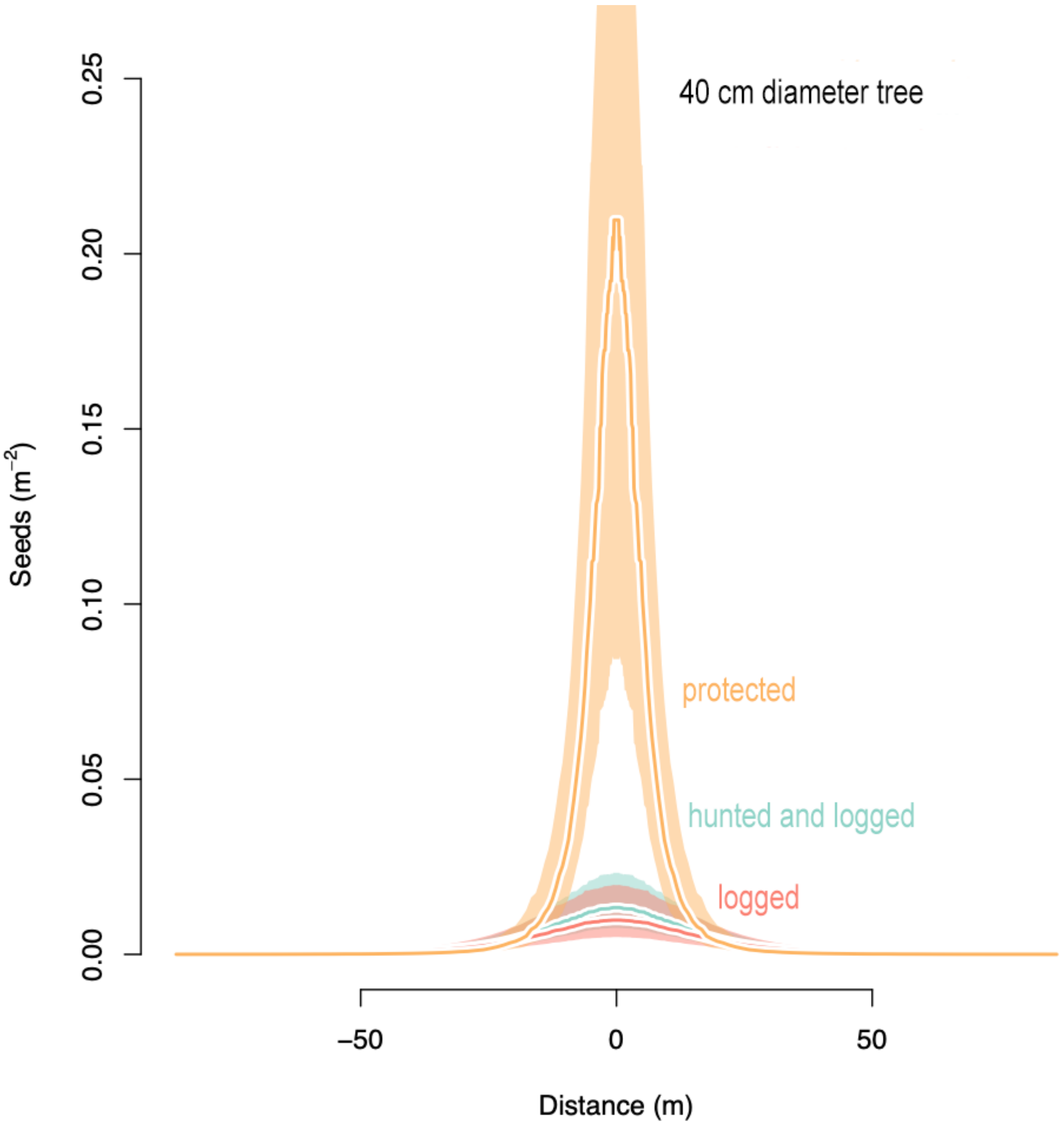

S6 (A-C): Examples of model diagnostics for *Nesogordonia kabingaensis*.

S6A: Trace plots of posterior chain convergence for all parameter values.

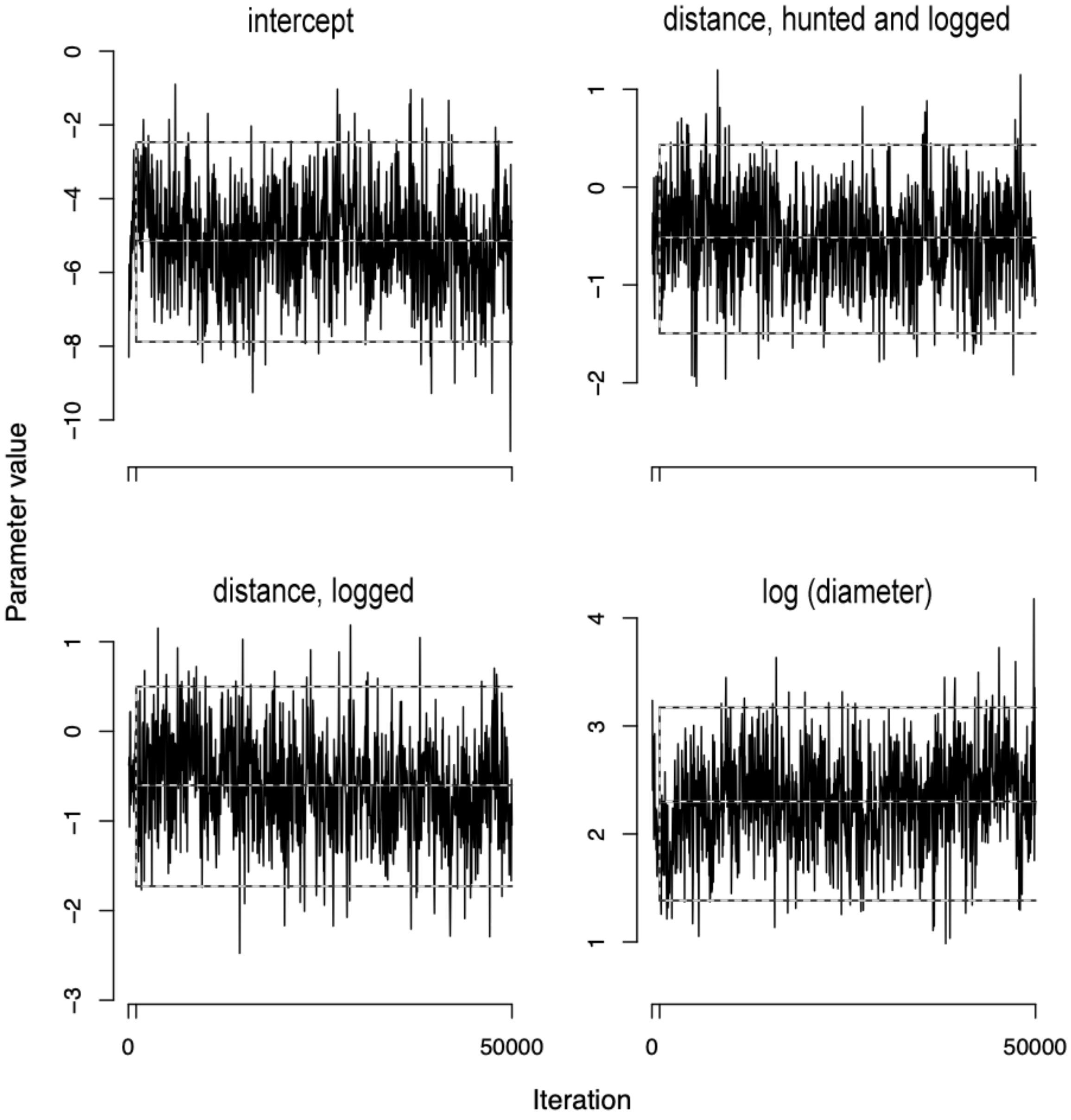

S6B: Trace plots of posterior parameter estimates of dispersal distance across disturbance regimes.

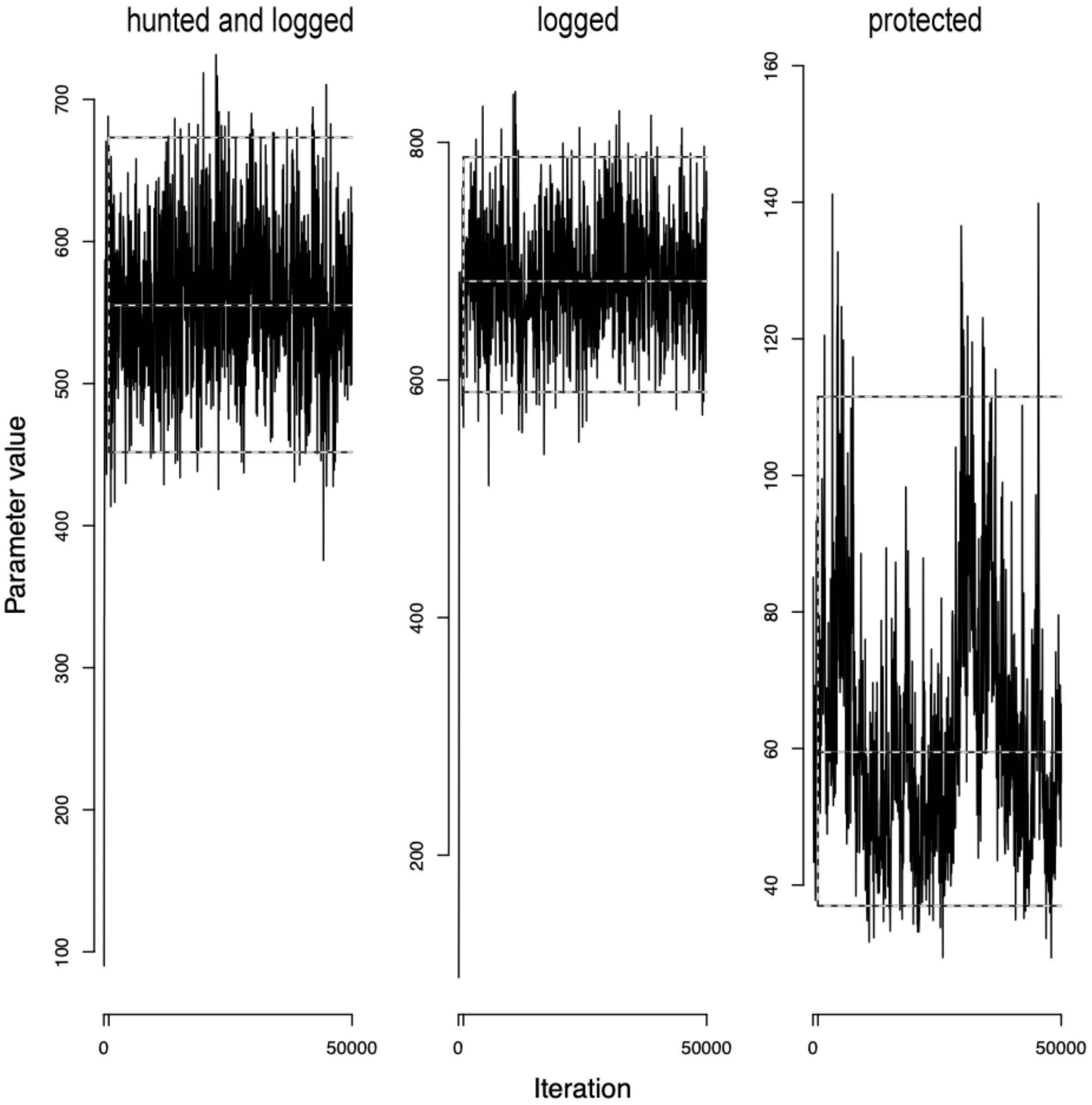

S6C: Trace plots of posterior parameter estimates of sigma, root mean squared prediction error (RMSPE), and deviance.

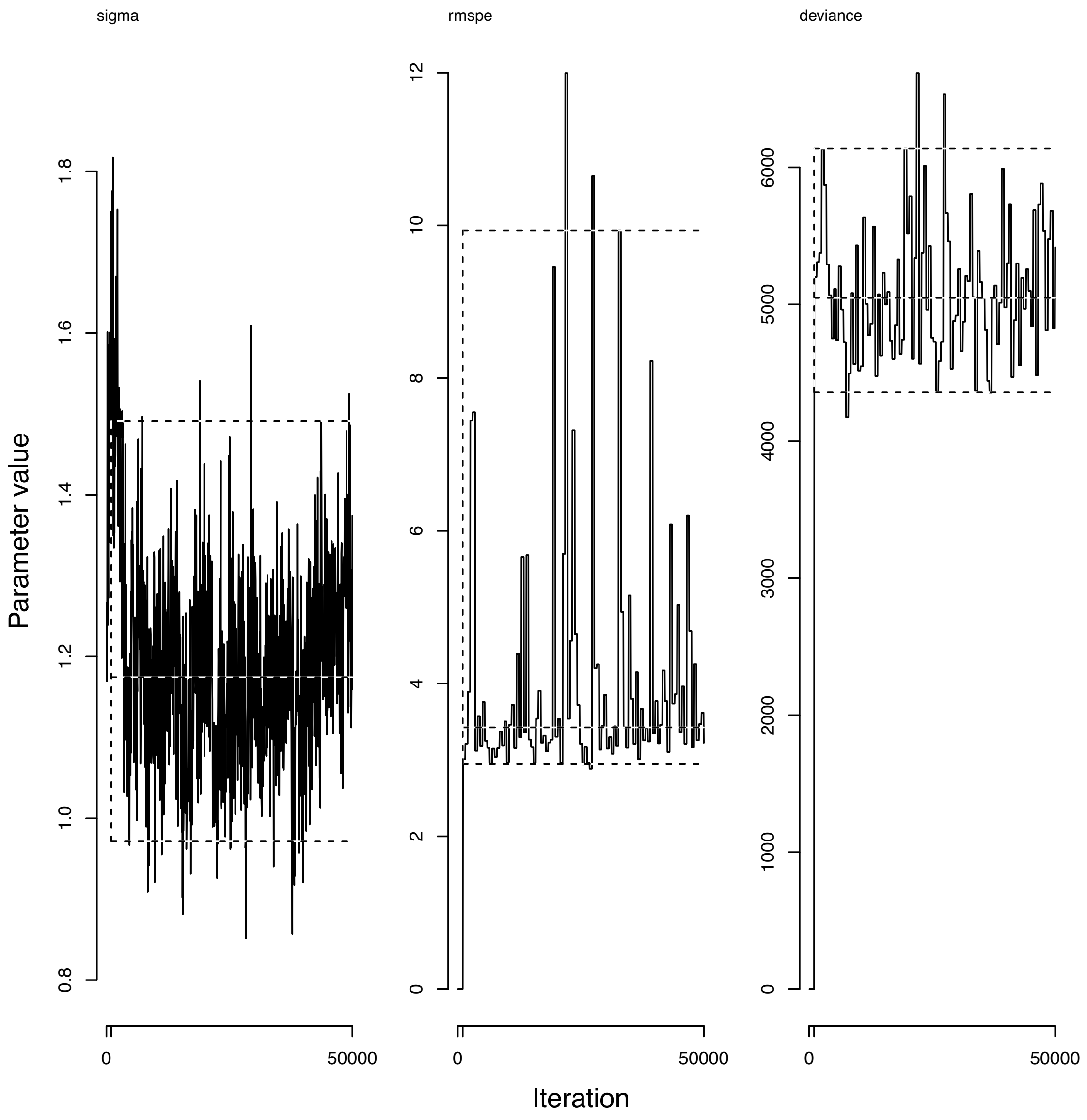

Table S1: Table of species information and dispersal vectors

	Bird Dispersed?	Mammal Dispersed?	Timber Species?
**Abiotically Dispersed**			
Albizia gummifera	no	no	no
Erythrophleum suaveolens	no	no	yes
Nesogordonia kabingaensis	no	no	yes
Petersianthus macrocarpus	no	no	yes
Pteleopsis hylodendron	no	no	yes
Pterocarpus soyauxii	no	no	yes
Terminalia superba	no	no	yes
**Animal Dispersed**			
Angylocalyx pynaertii	no	yes	yes
Celtis adolfi-friderici	no	yes	yes
Celtis mildbraedii	yes	yes	yes
Cleistopholis patens	yes	yes	yes
Diospyros bipindensis	no	yes	yes
Diospyros canaliculata	no	yes	yes
Greenwayodendron suaveolens	yes	yes	yes
Guarea cedrata	yes	yes	yes
Guarea thompsonii	yes	no	yes
Lannea welwitschii	yes	yes	yes
Macaranga barteri	yes	yes	yes
Staudtia kamerunensis	yes	yes	yes
Strombosia nigropunctata	yes	yes	yes
Strombosia pustulata	yes	yes	no
Strombosiopsis tetrandra	yes	yes	yes
Xylopia chrysophylla	yes	yes	no
Xylopia hypolampra	yes	yes	no
Xylopia phloiodora	yes	yes	no
**Abiotic & Animal Dispersed**			
Camptostylus mannii	no	yes	no
Grossera macrantha	no	yes	no
Lepidobotrys staudtii	yes	yes	no
Myrianthus arboreus	no	yes	no
Pausinystalia macroceras	yes	yes	yes
Radlkofera calodendron	yes	yes	yes
Rinorea oblongifolia	no	yes	yes
Thomandersia hensii	no	no	yes
